# *APOE* and *TREM2* regulate amyloid responsive microglia in Alzheimer’s disease

**DOI:** 10.1007/s00401-020-02200-3

**Published:** 2020-08-25

**Authors:** Aivi T. Nguyen, Kui Wang, Gang Hu, Xuran Wang, Zhen Miao, Joshua A. Azevedo, EunRan Suh, Vivianna M. Van Deerlin, David Choi, Kathryn Roeder, Mingyao Li, Edward B. Lee

**Affiliations:** 1 Translational Neuropathology Research Laboratory, Department of Pathology and Laboratory Medicine, Perelman School of Medicine at the University of Pennsylvania, PA, USA.; 2 Department of Biostatistics, Epidemiology and Informatics, Perelman School of Medicine at the University of Pennsylvania, PA, USA.; 3 Department of Information Theory and Data Science, School of Mathematical Sciences and LPMC, Nankai University, Tianjin, China.; 4 School of Statistics and Data Science, Key Laboratory for Medical Data Analysis and Statistical Research of Tianjin, Nankai University, Tianjin, China.; 5 Department of Statistics and Data Science, Carnegie Mellon University, PA, USA.; 6 Graduate Group in Genomics and Computational Biology, Perelman School of Medicine at the University of Pennsylvania, PA, USA.; 7 Department of Pathology and Laboratory Medicine, Perelman School of Medicine at the University of Pennsylvania, PA, USA; 8 Heinz College of Public Policy and Information Systems, Carnegie Mellon University, PA, USA; 9 Computational Biology Department, Carnegie Mellon University, PA, USA

**Keywords:** APOE, TREM2, microglia, Alzheimer’s disease, transcriptomics, snRNA-seq

## Abstract

Beta-amyloid deposition is a defining feature of Alzheimer’s disease (AD). How genetic risk factors, like *APOE* and *TREM2*, intersect with cellular responses to beta-amyloid in human tissues is not fully understood. Using single-nucleus RNA sequencing of postmortem human brain with varied *APOE* and *TREM2* genotypes and neuropathology, we identified distinct microglia subpopulations, including a subpopulation of CD163-positive amyloid-responsive microglia (ARM) that are depleted in cases with *APOE* and *TREM2* risk variants. We validated our single-nucleus RNA sequencing findings in an expanded cohort of AD cases demonstrating that *APOE* and *TREM2* risk variants are associated with a significant reduction in CD163-positive amyloid-responsive microglia. Our results showcase the diverse microglial response in AD and underscore how genetic risk factors influence cellular responses to underlying pathologies.

## Introduction

Alzheimer’s disease (AD) is a progressive and fatal neurodegenerative disease that affects more than 40 million people worldwide and remains the leading cause of dementia in the elderly [1]. Neuropathological hallmarks include intracellular hyperphosphorylated tau aggregates and extracellular β-amyloid plaques, which coincide with activation of innate immunity, synaptic dysfunction, and neuronal loss [2–5]. Genome-wide association studies (GWAS) have identified >30 AD genetic risk loci, many of which appear to be related to innate immunity and microglial function, including *APOE* and *TREM2* variants, which are associated with high genetic risks for sporadic AD [6–10]. The *TREM2* R47H variant is associated with an approximately 3-fold increased risk for AD, while the *APOE* E4 variant is associated with a ~3- to 4-fold increased risk with one copy and ~10- to 12-fold increased risk with two copies [8, 11, 12].

Triggering receptor expressed on myeloid cells 2 (TREM2) is encoded by the AD risk gene *TREM2* and is a single pass transmembrane receptor that, upon ligand binding, activates a series of downstream intracellular signaling cascades linked to immune function [13]. TREM2 expression is highly specific to microglia within the central nervous system (CNS), and as such has been studied in various mouse models. Microglia isolated from mice lacking *Trem2* show deficient phagocytic functions and perturbed lipid metabolism [14].

*APOE* encodes apolipoprotein E, a 33–37 kDa glycoprotein that is produced by hepatocytes, adipocytes, and macrophages outside of the CNS and affects cellular cholesterol content and lipoprotein metabolism by way of cholesterol efflux and reverse cholesterol transport [15, 16]. In the brain, APOE is commonly expressed by astrocytes and, to a lesser extent, microglia and may be similarly involved in lipid transport as well as synaptogenesis within the CNS [16–19]. Three common isoforms of APOE exist as APOE E2, E3, and E4 which demonstrate altered structure and function [20]. Importantly, deletion of *Apoe* in mice blocks the main response of microglia to beta-amyloid [21, 22].

While *APOE* and *TREM2* have been shown to influence microglial phenotype and activation states in mice [21, 23], how genetic risk in human disease translates to cellular responses to beta-amyloid deposition in human tissues is not completely characterized. In this study, we used single-nucleus RNA sequencing (snRNA-seq) of human tissue to characterize microglial responses to Alzheimer disease neuropathologic change as a function of *APOE* and *TREM2* risk genotypes. We use novel bioinformatics approaches to identify and characterize four different microglial subpopulations including amyloid responsive microglia (ARM). Moreover, we found that *APOE* and *TREM2* risk variants were associated with the loss of ARM in the human brain, highlighting how genetic risk factors appear to converge on microglial cellular responses to human pathology.

## Materials and Methods

### Nuclei isolation from frozen human brain tissue

Fifteen samples with varying genotypes and pathologies, but matched for age and sex, were selected from the Center for Neurodegenerative Research (CNDR) Brain Bank at the University of Pennsylvania [24] (Table S1). Frozen middle frontal neocortex brain tissue was transferred to a pre-chilled dounce homogenizer. Homogenization buffer consisted of 0.25M sucrose in TKM buffer (50mM Tris-HCl, pH 7.5, 25mM KCl, 14mM MgCl_2_, and 0.4U/uL RNAse inhibitor (Promega)) and was added to the dounce homogenizer, at a ratio of 2 mL buffer per 125 mg of tissue. The homogenate was mixed with 2.3 M sucrose + TKM solution to generate a 1.6 M sucrose solution in a chilled ultracentrifuge tube pre-filled with 3 mL cushion buffer (2.3 M sucrose in TKM) and spun at 100,000 × g for 45 min at 4°C in a swinging bucket rotor (Sw-41Ti rotor on an XPN-80 ultracentrifuge). After ultracentrifugation, the myelin layer was removed, the supernatant aspirated and discarded, and the remaining pellet was re-suspended in 1X PBS and RNAsin Plus RNAse inhibitor. The samples were kept on ice for 5 minutes, passed through a Bel-Art FlowMi 40um tip strainer, and then counted on a hemocytometer.

### Single nucleus RNA sequencing

Nuclei were sequenced using droplet-based single-nucleus RNA sequencing (snRNA-seq) using the Chromium system (10x Genomics) to generate libraries for sequencing on the Illumina HiSeq 2500 platform as per manufacturer’s instructions by the Center for Applied Genomics Sequencing Core at the Children’s Hospital of Philadelphia.

### Quality control

The raw fastq files were aligned to the hg38 genome sequence (GRCh38.p12 NCBI: GCA_000001405.27) using CellRanger (v.3.0.2). The snRNA-seq captures both unspliced pre-mRNA as well as mature mRNA. To include intronic reads from unspliced pre-mRNA, we generated a ‘pre-mRNA’ reference and obtained gene counts that included both exonic and intronic reads following steps recommended by 10x Genomics: https://support.10xgenomics.com/single-cell-geneexpression/software/pipelines/latest/advanced/references.

After alignment and gene counting, all 15 individual samples were merged into a single dataset with a median value of 845 genes and a median value of 1,211 UMI counts. In total, we detected 146,437 nuclei including 33,538 (29,435) genes across 15 samples in the initial dataset. The number of nuclei in each individual sample ranges from 5,526 (Sample 11) to 13,717 (Sample 8) and the median is 11,084 (Sample 1, Table S2).

Before further analysis, nuclei with high RNA content, which are likely to be doublets, were removed from the dataset. For each nucleus, the following criteria was used for filtering: a nucleus was kept if i) the number of expressed genes is between 200 and 4,000 and ii) mitochondrial gene UMI proportion is less than 5%. Genes expressed in less than 10 cells were removed from further analysis. After filtering, the final dataset included 26,423 genes profiled in 131,239 nuclei. The number of nuclei in each individual sample ranges from 2,838 (Sample 11) to 13,649 (Sample 8) and the median is 10,492(Sample 4, Table S2).

### Preprocessing

Before clustering, the gene expression profiles were normalized using SCANPY 1.4.2 [25]. Within each nucleus, the UMI count for each gene was divided by the sum of UMI counts across all genes and then multiplied by 10,000, using function *normalize_per_cell* with parameter counts_per_cell_after = 10000. The log transformation was performed on the normalized expression values using function *log1p*. Next, we selected 2,000 highly variable genes using function *highly_variable_genes* with parameter n_top_genes = 2000. The expression values of highly variable genes were scaled to unit variation with zero mean for each sample.

### Clustering

The 131,239 nuclei were clustered based on the 2,000 highly variable genes using DESC, an algorithm that can remove batch effect effectively. DESC is an unsupervised deep embedding algorithm for single-cell clustering. It projects the gene expression profiles to a low dimensional non-linear latent space by an autoencoder network. Then the network is connected to a cluster layer to perform iterative clustering. Batch effect is removed simultaneously during the iterative clustering procedure. We used two hidden layers for encoder with 256 nodes in the first layer and 128 nodes in the second layer. The cluster centers were initialized by Louvain’s clustering algorithm, which is a graph-based community detection algorithm [26]. The resolution parameter of the Louvain algorithm affects the number of the clusters. In general, lower resolution leads to fewer clusters and a higher resolution leads to more clusters.

To identify an optimal resolution parameter, we performed the DESC analysis 15 times using different resolutions ranging from 0.1 to 0.8 with an incremental 0.05 step. The number of clusters ranged from 8 to 16. Fig. S2 shows the clustering results from 4 selected resolutions [0.2, 0.4, 0.6, 0.8]. The t-SNE plot was colored by cluster ID, total UMI counts, maximum probability for cluster assignment, and sample ID, respectively. We found that some clusters expressed more RNA transcripts (e.g. neurons) than other clusters (e.g. non-neuronal cells). The maximum probability for cluster assignment is output from DESC. After clustering, DESC assigns a probability for a cell belonging to a cluster. Each cell was colored by maximum cluster assignment probability. A higher maximum probability means higher confidence for cluster assignment. Our results indicate that most nuclei were clustered with a high maximum probability. The t-SNE plot colored by sample ID shows that the nuclei were randomly mixed across different samples, indicating that the batch effect was appropriately removed in clustering.

### Batch effect analysis

To evaluate whether batch effect was successfully removed during clustering, we calculated the Kullback–Leibler (KL) divergence, also called relative entropy, to examine how randomly cells from different batches (i.e., samples) were mixed together. The KL-divergence of batch mixing for *B* different batches was calculated as $KL=\sum_{b=1}^{B}p_{b}log=\frac{p_{b}}{q_{b}}$, where *q*_*b*_ is the proportion of cells from batch *b* among all cells, and *p*_*b*_ is the proportion of cells from batch *b* in a given region based on results from a clustering algorithm, with $\sum_{b=1}^{B}q_{b}=1$ and $\sum_{b=1}^{B}p_{b}=1$. We calculated the KL divergence of batch mixing by using regional mixing KL divergence defined above using *N* randomly chosen cells from all batches. The regional proportion of cells from each batch was calculated based on the set of *K* nearest neighbor cells from each randomly chosen cell. Smaller KL divergence indicates better batch mixing, i.e., more effective in batch effect removal in clustering.

We first set *N* = 100 and *K* = 100 to calculate the KL divergence for all clustering results obtained from 15 resolutions. The distances between cells were calculated based on the embedding space output by DESC. Fig. S2(B) shows that the KL divergence is stable across 15 resolutions. The median KL divergence of each clustering result ranges from 0.23 to 0.32. The small range of KL divergence suggests that DESC clustering results are robust to batch effect. Next, we varied the value of *K* = [100, 200, 300, 400, 500, 1000, 2000, 3000, 4000, 5000] when *N* = 100 and the embedding space was obtained when resolution was set at 0.15. As expected, Fig. S2(C) shows that the KL divergence decreases as *K* increases; the median KL divergence decreased from 0.30 to 0.13 as *K* increased from 100 to 5000.

To further evaluate if DESC is effective in removing batch effect, we compared with KL divergence obtained based on Euclidean distance of the top 2000 highly variable genes selected from the original expression data after they were normalized and scaled. In this situation, the median values for KL divergence are 0.85 (*K* = 100) and 0.29 (*K* = 5000), which are much larger than those obtained from DESC, suggesting that DESC is effective in removing batch effect.

### Cell type annotation

After clustering using a given resolution parameter, the resulting clusters were annotated using known marker genes for 7 cell types, including microglia, endothelial cells, excitatory neurons, inhibitory neurons, astrocytes, oligodendrocytes and oligodendrocyte progenitor cells (Fig.S3). Since we clustered the data using 15 resolutions in DESC, each nucleus has 15 cell type labels, one for each resolution. A consensus step was applied to get the final cell type annotation. A nucleus was labeled as one of the seven cell types if its 15 cell type labels were the same for all resolutions. This stringent cell type annotation procedure worked well for most nuclei except endothelial cells and oligodendrocyte progenitor cells. The reason is that endothelial cells are much less abundant than other types of cells (444 nuclei only) and oligodendrocyte progenitor cells are similar to oligodendrocytes. These two types of cells were mixed with oligodendrocytes in the partition of resolution 0.1. Due to this reason, endothelial and oligodendrocyte progenitor nuclei were annotated if a nucleus had the same cell type labels in 14 resolutions. Nuclei that could not be annotated using this consensus procedure were removed from further analysis. This left 122,606 well annotated nuclei, which included 3,982 microglia, 444 endothelial, 39,176 excitatory neurons, 12,286 inhibitory neurons, 15,683 astrocytes, 44,182 oligodendrocytes and 6,853 oligodendrocyte progenitor cells. Table S3 shows the number of nuclei for different cell types across samples. The proportion of cell types across different genotypes (*TREM2*, *APOE*) and pathology (AT score) are shown in Fig. S4.

### Identification of microglia subpopulations

To identify microglia subpopulations, we re-clustered the microglia cells using DESC. We selected 5,000 highly variable genes in order to detect subtle cellular differences among the microglia. A single 128-node layer was applied for the encoder in DESC and the resolutions ranged from 0.1 to 0.8 with an incremental 0.05 increasing step. The numbers of clusters ranged from 2 to 7 with the 15 resolutions. Since the clustering results did not change much when the resolution parameter was greater than 0.6, we selected the clustering result with a resolution of 0.8 as the final cluster result.

The microglia cells were grouped into 7 subpopulations. Although we used a consensus procedure to annotate cells, upon further examination, we found that three of the subpopulations might be contaminants because they express other marker genes for other cell types (oligodendrocytes, astrocytes and neurons). Therefore, we labeled these three subpopulations as Oli_microglia, Ast_microglia and neuronglia, respectively. They were considered as contaminations and removed from further analysis. The other four subpopulations did not express other marker genes and are close to each other after projecting them onto a 2D t-SNE plot. Based on pathology and genotype information in these subpopulations, we annotated them as homeostatic microglia, amyloid responsive microglia (ARM), motile microglia and dystrophic microglia. These four subpopulations include 2,773 nuclei in total, and Table S4 shows the number of nuclei in each subpopulation across samples.

### Microglia contamination

Contaminated microglia were identified by checking marker gene expression for other cell types. We found that cells in the Oli_Microglia cluster were enriched for *MBP*, *MOG* and *OLIG1*, known marker genes for oligodendrocytes; cells in Ast_Microglia were enriched for *GFAP*, *ALDH1L1*, *AQP4* and *SLC1A2*, known marker genes for astrocytes; and cells in Ex_Microglia were enriched for excitatory neuron marker genes *SLC17A7*, *GRIN1* and *GRIN2B* (Fig.S5). These three subpopulations were considered as contaminated by oligodendrocytes, astrocytes, and excitatory neurons, respectively. Additionally, cells in these three subpopulations tended to have more expressed genes and RNA content than cells in the other four subpopulations (Fig.S5).

For further confirmation, we clustered the microglia cells in our dataset with the Microglia_Macrophage and ImOLG cells reported in the Jakel *et al.* dataset [27]. This paper identified an oligodendrocyte subpopulation named ImOLGs because it was closely associated with microglia and expressed genes such as innate immune activation genes like *CD74*, *HLA-DRA*, *PTPRC* and *C3*. To assess whether immune oligodendrocytes were a contaminant within the microglial populations, UMI expression and cell type annotation tables of the Jakel *et al.* dataset was download from GEO (accession number GSE118257). This dataset includes 428 Microglia_Macrophage cells and 207 ImOLG cells. We merged our microglia data and the Jakel *et al.* data by genes names. The gene expression values were scaled to mean zero and one unit separately for these two datasets.

Clustering analysis was performed on the merged dataset using DESC with the top 5000 highly variable genes and a single 128-node encoder layer and resolutions from 0.6 to 2.0. Figure S5 (C) shows the t-SNE plot of clustering result when resolution was set at 1.4. We found that the Oli_Microglia subpopulation was mixed together with the ImOLGs cells and the four subpopulations without contamination were mixed together with Microglia_Macrophage cells. These results further confirmed that Oli_Microglia expressed microglia and oligodendrocyte markers simultaneously. Although Oli_Microglia was possibly contaminated by oligodendrocytes, we cannot completely rule out the possibility that this subpopulation might be genuine subpopulation. Further investigation is needed to have a better understanding on the role of this subpopulation.

### Differential expression analysis

Differential expression (DE) analysis for the microglia subpopulations was performed using SCANPY 1.4.2. Significant DE genes were identified based on Wilcoxon rank-sum test with a Benjamini-Hochberg corrected *P* value < 0.05 and the log2 fold change greater than 0.1. The DE analysis was performed using function *rank_genes_groups* with parameters method = ‘wilcoxon’ in SCANPY.

We performed two different types of DE analysis. First, the ARM, motile and dystrophic subpopulations were compared to homeostatic microglia separately (i.e., one vs homeostatic). The number of upregulated significant DE genes are 72, 34 and 151 for ARM, motile and dystrophic vs homeostatic, respectively. The numbers of downregulated significant DE genes were 37, 20 and 22, respectively (Data S1). Second, each subpopulation was compared to the rest of the nuclei in the other three subpopulations (i.e., one vs rest). The number of upregulated significant DE genes were 28, 87, 109 and 127, respectively, for homeostatic, ARM, motile and dystrophic microglia. The numbers of downregulated significant DE genes were 41, 86, 150 and 10 (Data S2). Fig. S6(A) shows the volcano plots of DE genes for the four subpopulations in ‘one vs rest’ mode.

### GO enrichment analysis

We used the panther (pantherdb.org) server to perform statistical overrepresentation analysis for the DE genes to identify enriched gene ontology (GO) categories [28]. All genes of *homo sapiens* were used as background. The binomial test with FDR correction was applied to the Biological Processes ontology. A GO term was considered significantly enriched if the FDR adjusted *P* value was < 0.05.

The GO enrichment analysis was performed to two different sets of DE genes, ‘one vs homeostatic’ and ‘one vs rest’. The ‘one vs homeostatic’ GO enrichment terms are available in Data S3, and ‘one vs rest’ GO enrichment terms are shown in Fig. S6(B) and available in Data S4.

### Cell-Specific Network (CSN) and MetaCell analysis

Cell-specific-network (CSNs) are constructed by a method adopted from Dai *et al.* with modifications [29]. Starting from single cell RNA-seq data, Dai *et al*.’s method produces a gene-gene network for each cell, which indicates if there is an edge between genes. The modifications made include assigning non-expressed genes with no edges, since the connectivity involving non-expressed genes are difficult to determine purely by test statistics due to dropout events. Second, the test statistics were calculated with adapted window sizes, which were determined by the local standard deviation rather than a quantile. This allowed us to treat outlying and interior points with equal power when calculating the test statistics, thus producing a better representation of gene co-expression for each cell.

Due to the sparsity of expression, a direct application of the CSN algorithm failed to discover network structure, especially for small cells like microglia. Therefore, we applied the Metacell algorithm before constructing CSNs. Metacell partitions scRNA-seq datasets into metacells, defined as disjoint clusters of homogenous profiles [30]. After applying Metacell to pre-labeled single cell data, we further divided metacells with multiple cell types or subtypes into pure-cell-type metacells. Expression of a metacell is defined as the mean of the cells in the cluster, which alleviates the problem of having zero expression for many genes per cell. The metacells were then treated as cells for the purpose of constructing metacell-specific-networks.

Before forming metacells, we filtered genes to include only those with expression rate greater than 5% across all cells (7066 genes remaining). Then we applied the Metacell algorithm to the expression data of those 7066 genes. When Metacell filtered small cells by library size, we set the cut-off at 200 to preserve microglia cells, which are relatively small. All other parameters for Metacells were set at default values. Suggested by Dai et al., gene expression is measured by FPKM. To avoid batch effects, we applied Metacell and construct CSNs for each subject independently. In total we formed 17, 46, 43 and 20 metacells for microglia subtype homeostatic, motile, ARM and dystrophic, respectively, and, on average, there were approximately 50 cells in each metacell.

Next, we compared CSNs across microglia subtypes. CSNs can be viewed as random vectors and the differences are tested by a simplified version of a multivariate data change point test (Matteson and James, 2014).

Let *X*_1_, …, *X*_*k*_ denote the vectorized CSNs from one subtype and *Y*_*k*+1_, …, *Y*_*n*_ denote the vectorized CSNs from another subtype. This method makes following assumptions:

(A1) *X*_*i*_ and *Y*_*j*_ are independent from each other;
(A2) *X*_1_, …, *X*_*k*_ *iid* ~ *F*_1_ and *Y*_*k*+1_, …, *Y*_*n*_ *iid* ~ *F*_2_;
(A3) *E*|*X*_*i*_|^*α*^ < ∞ and *E*|*Y*_*j*_|^*α*^ < ∞ for some *α* ∈ (0, 2).

(A1) means that the CSNs from different subtypes are independent from each other and (A2) means that the CSNs from the same subtype are independent and follow the same distribution. These assumptions hold when CSNs are constructed from homogeneous subjects. Notice that this method is nonparametric and does not require specific form for distribution *F*_1_ and *F*_2_. Because CSNs contains entries that are either 0 or 1, (A3) naturally holds.

This non-parametric method viewed the sequence of vector *X*_1_, …, *X*_*k*_, *Y*_*k*+1_, … *Y*_*n*_ as a time series with known change point at time *k* and test null hypothesis *H*_0_: *F*_1_ = *F*_2_ versus alternative *H*_1_: *F*_1_ ≠ *F*_2_. The test statistic is a scaled alternative divergence measurement based on Euclidean distance (Eq 1) and the p-value is calculated by permutation test.

(Eq 1) $$\text{Q}\left(\text{X},\text{Y};\alpha \right)=\frac{2}{n}\sum_{i=1}^{k}\sum_{j=k+1}^{n}{\left|X_{i}-Y_{j}\right|}^{\alpha }-\frac{k\left(n-k\right)}{n}{\left(\begin{array}{c} k\\ 2 \end{array}\right)}^{-1}{\sum_{1\leq i<i^{\prime }\leq k}\left|X_{i}-X_{i^{\prime }}\right|}^{\alpha }-\frac{k\left(n-k\right)}{n}{\left(\begin{array}{c} n-k\\ 2 \end{array}\right)}^{-1}\sum_{k+1\leq j<j^{\prime }\leq n}{\left|Y_{j}-Y_{j^{\prime }}\right|}^{\alpha }.$$

### Cell-specific Network (CSN) heatmaps and Sankey plots

We restricted our CSN comparison between microglial subtypes to 477 differentially expressed genes, which were selected based on their p-values from an ANOVA test of FPKM measured expression across four microglia subtypes. With these 477 genes, we tested the differences across subtypes for all subjects by the multivariate data change point test and the unadjusted p-values (Table S5). There were significant differences between all pairs of subtypes, and the p-values confirmed the differences between microglia subtypes. The p-values calibrate that the differences between homeostatic and dystrophic as well as motile and ARM are smaller than that between other pairs of subtypes.

We also investigated the sets of genes that are transferred across gene clusters between subtypes using PisCES [31]. As input PisCES utilizes the average network for two subtypes and clusters genes by Laplacian smoothing and K-means. It also orders genes within clusters and infers how gene sets travel between subtypes. Gene connection within subtypes and transformation between subtypes are visualized by Sankey plots [31]. Heatmaps plot average CSNs for each subtype and the colors show strength of gene-gene connection, ranging from none (white) to strong (red). For each subtype, genes were ordered by PisCES and the order can be different between subtypes. The color bands connecting CSN heatmaps show the flow of major sets of genes from subtype to subtype.

### Trajectory analysis

Monocle 3 was employed for trajectory analysis on the 4 subpopulations of microglia [32]. We followed the Monocle 3 workflow to generate trajectories. Firstly, the data were preprocessed by *preprocess_cds* with parameter num_dim = 100 and method = ‘PCA’. Then dimensionality reduction was performed by function *reduce_dimension* with default parameters (UMAP method was applied). Next, nuclei in the reduced space were clustered by function *cluster_cells* with default parameters. Finally, Monocle 3 constructed the trajectories of nuclei using function *learn_graph* and estimated pseudotime using function *order_cells*. The Homeostatic subpopulation was selected as root when estimating the pseudotime.

We also applied partition-based graph abstraction (PAGA) to build trajectories among the microglia subpopulations [33]. PAGA generates graphic-like maps which preserves the global topology of the data. First, a kNN (k nearest neighbor, k = 20) graph was constructed to represent the gene expression data using PCA-based representation and Euclidean distance. Then we used the microglia subpopulations as partitions to get a PAGA graph via function *tl.paga* in SCANPY. The PAGA graph represents each subpopulation as a node and connects nodes with weighted edges, which represent a statistical measure of connectivity between subpopulations. To estimate pseudotime, PAGA applies an extended version of diffusion pseudotime (DPT), and the reference was via function *tl.dpt*. We picked a cell randomly from the homeostatic subpopulation as the root to infer pseudotemporal ordering.

### GWAS analysis

404 AD associated GWAS loci mapped genes were obtained from the GWAS Catalog together with 6 newly discovered genes from Kunkle et al. [10, 34]. We further required the genes to have non-zero UMI counts in at least 20% of microglia cells in at least one subpopulation, which resulted in a list of 32 AD associated genes. For each gene, the mean expression value in each group (microglia subcluster or AT score) was calculated and normalized to Z-scores as the input of the heatmap.

### Neuropathologic validation of snRNA-seq tissue

The 15 fresh frozen human brain tissue cases used for snRNA-seq were additionally fixed in 10% formalin for 48 hours and processed in a routine fashion [24]. The formalin fixed paraffin-embedded sections were then stained for beta-amyloid (NAB228) and phospho-tau (PHF1). Afterwards, AT scores, where presence of amyloid was designated A+ and tau tangles were designated T+, were assigned to each portion of tissue by two independent neuropathologists blinded to clinical and genotype data (Table S1).

### Case selection of expanded autopsy cohort and immunohistochemistry

Forty-eight cases were selected from the Center for Neurodegenerative Disease Research (CNDR) Brain Bank at the University of Pennsylvania based upon genotype and pathology [24]. Fifteen cases were first identified with *TREM2* R47H variants. Cases were excluded with additional mutations, variants, or non-amyloid or non-tau co-pathology in the neocortex. Control *TREM2* WT cases were selected to match pathology, *APOE* allele genotype, sex, and age (Table S1).

Single immunohistochemistry (IHC) was performed on 6 or 20 μm formalin- or ethanol-fixed, paraffin-embedded tissue sections from the middle frontal or angular neocortex. After deparaffinization and rehydration of the tissue, sections were treated with methanol/H_2_0_2_ for 30 minutes and then washed for another 10 minutes. Microwave antigen retrieval in citric acid-based antigen unmasking solution (Vector Laboratories, Burlingame, CA, USA) was then performed. Sections were washed in 0.1 M Tris buffer and blocked in 2% fetal bovine serum (FBS) in 0.1 M Tris buffer. Sections were then incubated with primary antibodies (Table S6) overnight at 4°C in a humidified chamber. Sections were again washed in 0.1 M Tris buffer, blocked in 2% FBS in 0.1 M Tris buffer, and then incubated with species-specific antibodies for 1–2 hours at room temperature. Afterwards, sections were once more washed and blocked and incubated with AB solution (Vectastain ABC kit, Vector Laboratories, Burlingame, CA, USA) for 1.5 hrs at room temperature. Sections were once more washed and developed using DAB (3,3′-diaminobenzidine) peroxidase substrate kit (Vector Laboratories, Burlingame, CA). Lastly, sections were counterstained with Harris’s hematoxylin (Shandon Harris Hematoxylin, Thermo Scientific, Cheshire, WA, USA), dehydrated in an ascending ethanol series, and cleared using xylene. Mounting media (Cytoseal TM 60, Thermo Scientific, Cheshire, WA, USA) and glass coverslips (Fisherbrand Microscope Cover Glass, Pittsburgh, PA, USA) were used to coverslip the slides.

Briefly, for dual immunohistochemistry, formalin-fixed, paraffin-embedded sections were processed as aforementioned and stained using an anti-CD163 antibody (Table S5) and developed using DAB, while beta-amyloid plaques were stained using an anti-amyloid antibody (Table S5) and developed using the VECTOR Red Alkaline Phosphatase (Vector Laboratories, Burlingame, CA, USA) detection kit.

### Digital Image Analyses

For digital image analysis, slides stained for CD163, amyloid (NAB228), and PU.1 were scanned at 20x magnification by the Lamina (Perkin Elmer, Waltham, MA) slide scanning system. Images were then analyzed using QuPath v0.2.0-m2 [35] where the cortex for each case was manually annotated as the region of interest (ROI), and the positive thresholds for hematoxylin and DAB were set for each case in a blinded manner.

Percent positive area was calculated for CD163 and amyloid (NAB228) using the positive pixel count feature under region identification, and annotation measurements of percent area were exported as txt files. For PU.1 nuclei staining, after manual annotation of the cortex and thresholding values of hematoxylin and DAB, a custom script was written and employed to calculate positive cell detection under cell analysis. The results of absolute number of PU.1 positive and negative nuclei were exported as txt files. All traces and annotations were manually re-checked in a blinded fashion to ensure threshold value robustness and accuracy.

## Results

### Single-nucleus RNA sequencing of postmortem human tissue reveals distinct CNS populations

**Fig. 1:**
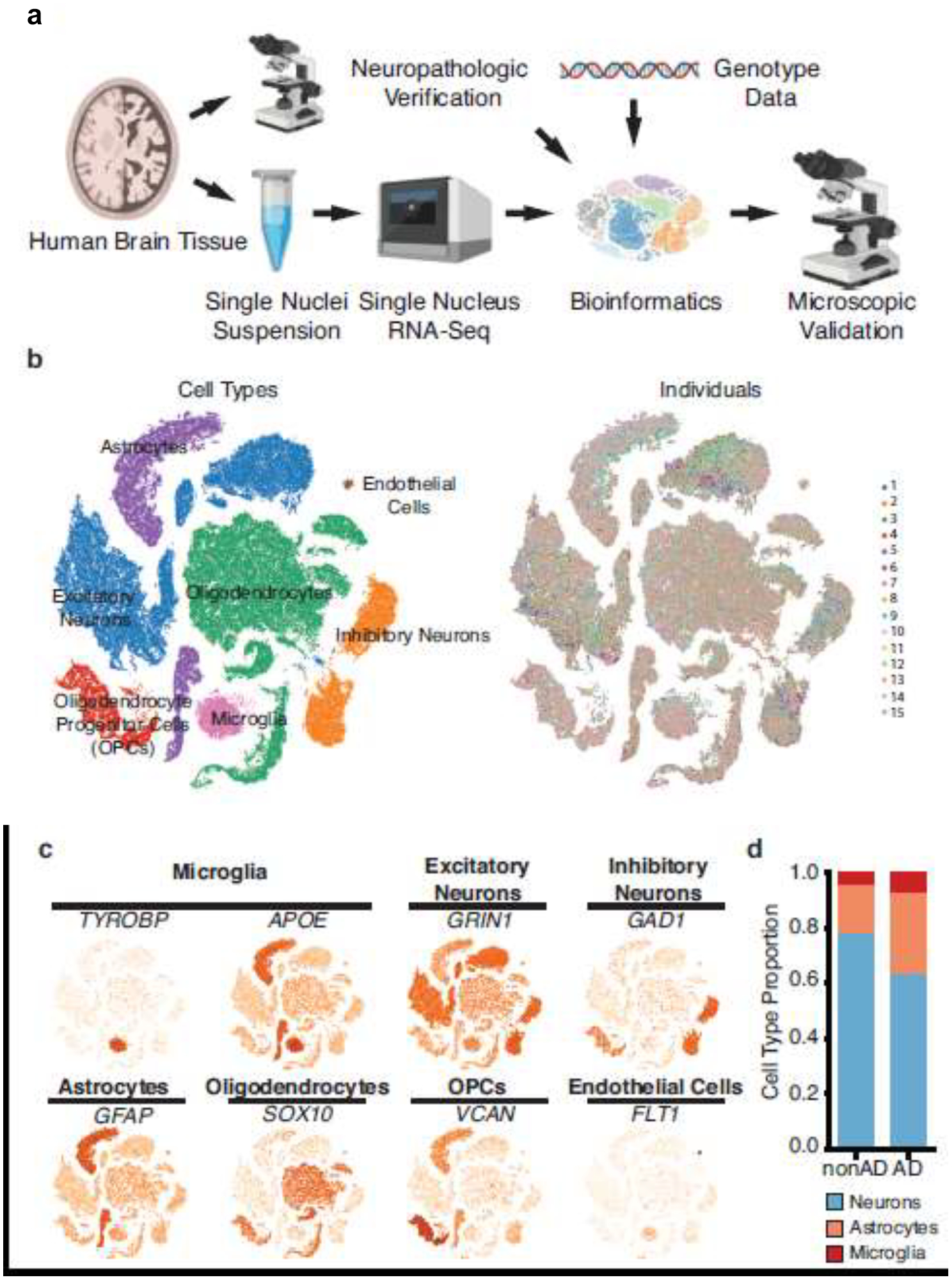
(a) Schematic of the analysis pipeline. (b) The left figure is a t-distributed stochastic neighbor embedding (t-SNE) projection of all cells (n=122,606 from 15 brains) after quality control filtering (Table S2 and S3): *Microglia(Mic); Excitatory neuron(Ex); Inhibitory neuron(In); Astrocytes(Ast); Oligodendroctyes (Oli); Oligodendrocyte progenitor cells (Opc); Endothelial (End)*. The right figure is a t-SNE plot colored by 15 samples. (c) Expression feature plots of known cell type-specific marker genes. (d) The proportions of neurons (excitatory and inhibitory neurons), astrocytes, and microglia across cases without or with AD neurofibrillary degeneration

Consistent with the proposed biological definition of AD [36] and due to the known variability of AD neuropathology from region to region, human dorsolateral prefrontal cortex tissue specimens were scored neuropathologically for amyloid and tau pathologies as an “AT” score (Table S1) to obtain a series of annotated tissues with variable AD neuropathology. Cases were also genotyped for *APOE* and *TREM2* risk variants. Nuclei were isolated from 15 cases for snRNA-seq using the 10x Genomics platform (Fig. 1A). 131,239 single nuclei gene expression profiles were generated, with a median of 850 genes and 1,445 transcripts per nucleus (Fig. S1). A novel machine learning method known as deep embedding algorithm for single-cell clustering (DESC) [37] was utilized to ensure accurate clustering through iterative learning. After serial clustering at various resolution parameters (Fig. S2), seven cell type clusters emerged at the optimum DESC resolution allowing for accurate classification of nearly all nuclei (n=122,606; Fig. 1B, left). Nuclei from each case were admixed with each other within cell clusters (Fig. 1B, right), demonstrating that DESC removes batch effect and technical variability artifacts. Known cell-type-specific markers were used to annotate clusters allowing for identification of microglia, excitatory neurons, inhibitory neurons, astrocytes, oligodendrocytes, oligodendroglial precursors, and endothelial cells (Fig. 1C, Fig. S3, and Table S3). Tissues with neurofibrillary degeneration (A+T+) demonstrated fewer neurons with a concomitant expansion of reactive glia relative to tissues without neurofibrillary tangles (A+T− or A−T−; Fig. 1D and Fig. S4).

### Transcriptional, pathologic and genotypic heterogeneity in microglial subpopulations

**Fig. 2:**
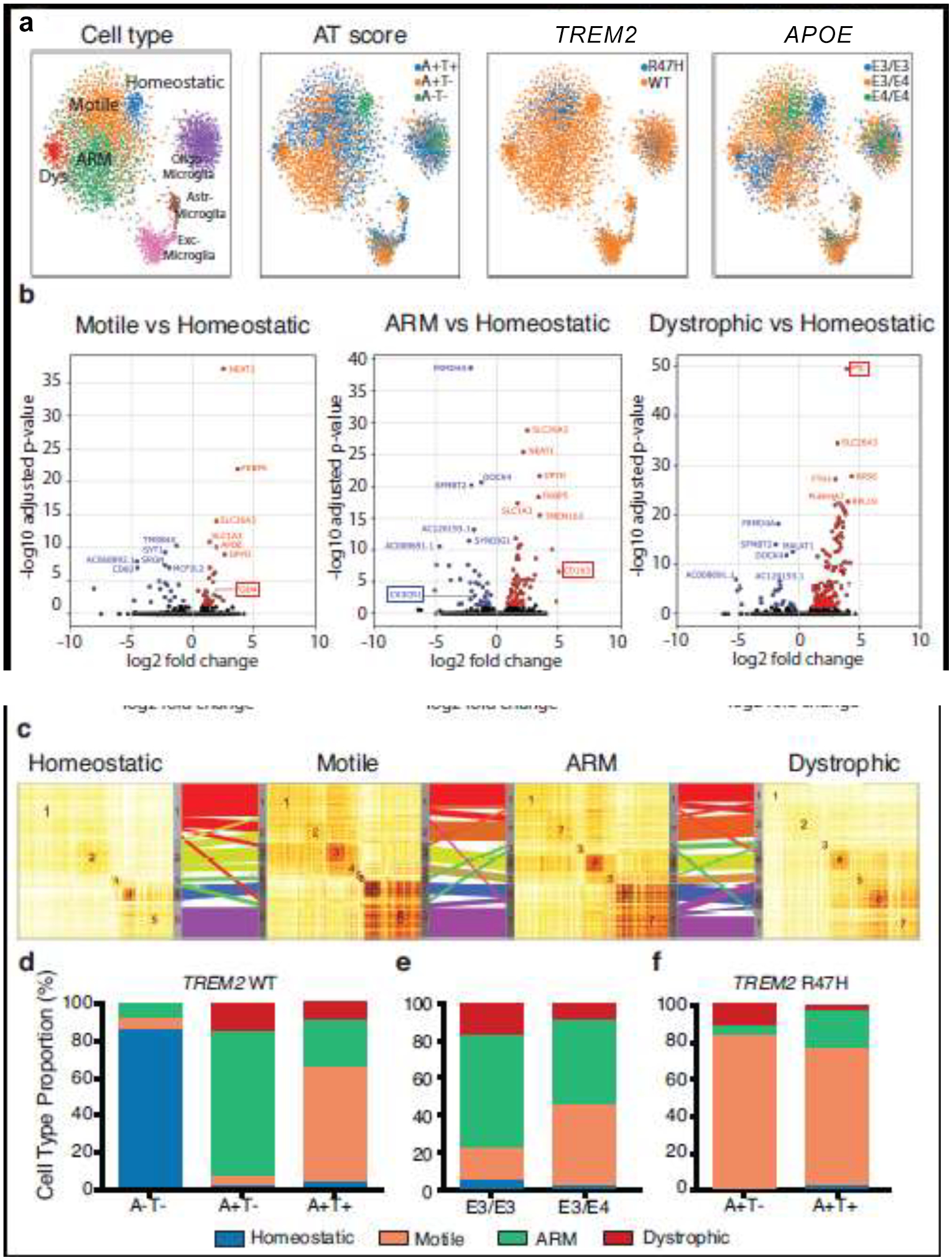
Microglia subclusters are unique by pathologic characterization, differentially expressed genes, genotype, or co-expression network analysis. (a) t-SNE projections of microglia subpopulations additionally colored by cell type, AT score, *TREM2* R47H genotype, and *APOE* genotype. (b) Differentially expressed genes (DEGs) between subpopulations: motile vs homeostatic, ARM vs homeostatic, and dystrophic vs homeostatic. The genes in red are upregulated and those in blue are downregulated. Gene markers of interest per subcluster are highlighted with boxes. (c) Co-expression modules for each microglia subtype depicting gene-gene co-expression networks, colored by the strength of the gene-gene connection ranging from none (white) to strong (red). Intervening Sankey plots depict the flow of gene clusters from one subtype to the next, wherein the width demonstrates the magnitude of flow. Only major flows with more than 12 genes are plotted. (d) Bar plots of the differing microglia subpopulations (homeostatic, dystrophic, motile and ARM) in *TREM2* WT cases with varying amyloid and tau pathology (AT scores A−T−, A+T−, and A+T+; *n*=2, 4, and 5 respectively; Fisher’s exact test performed for each pair-wise comparison) or (e) separated by *APOE* alleles, matched for pathology (*n*=2 for E3/E3 and *n*=6 for E3/E4; Fisher’s exact test performed for ARM proportion calculation). (f) Cell type proportions in *TREM2* R47H variant cases only, subdivided by pathologic classification (Fisher’s exact test performed for ARM proportion calculation compared to *TREM2* WT A+T+ cases). Note that only one case with the *TREM2* R47H variant and an A+T− score was available.

DESC was used to subcluster the 3,982 microglia which revealed four microglial subpopulations along with three smaller, separate clusters (Fig. 2A and Table S4). The smaller clusters showed both microglial transcripts and oligodendroglial, astrocytic, or neuronal transcripts, respectively. This might be attributed to artefactual instances of sequencing two nuclei formed from nuclei fusion during homogenization or library generation and not from the presence of hybrid cell types such as immune oligodendroglia (Fig. S5) [27]. The majority of the remaining nuclei (n=2,773) comprising the four microglial subclusters were designated based upon pathologic characterization or differentially expressed genes (DEGs). One microglial subcluster expressed high levels of the canonical homeostatic marker *CX3CR1* and was associated with normal tissues (A−T− pathologic classification) and thus designated as homeostatic microglia (Fig 2A and Data S1–S2). Another cluster designated as motile microglia showed a modest number of uniquely upregulated DEGs in comparison to the homeostatic population (Fig. 2B and Data S1), some of which are associated with cell motility, actin remodeling, and extracellular matrix remodeling (*ARHGAP15, ARHGAP24, BMP2K, CSGALNACT1, FGD4, FOXP1, N4BP2L2, and USP39*). In contrast, the final two microglial subclusters significantly overexpressed pro-inflammatory related genes (*C1QA, C1QB, FCGBP, FCGR1A, PAG, SKAP2, SLC11A1, and SPP1*). One of these clusters was designated dystrophic microglia (Fig. 2B) based on the marked expression of *FTL* and *FTH1* (Data S1–S2), which are known markers of dystrophic microglia [38, 39]. By gene-ontology enrichment analyses, dystrophic microglia are characterized by alterations in mRNA and RNA catabolism, SRP-dependent co-translational protein targeting to the membrane, nuclear transcription, translation, and transport (Fig. S6 and Data S3). The final microglial subcluster was designated amyloid-responsive microglia (ARM) as they were associated with the A+T− pathologic classification (Fig. 2A). Compared to homeostatic microglia, the highest upregulated DEG was *CD163*, a hemoglobin-haptoglobin receptor involved with both iron metabolism and inflammation, which was unique to this subcluster (Fig. 2B) [40]. Using gene ontology enrichment analyses, over represented terms for ARM were associated with responses to and sensing of stress, oxidation, and extracellular stimuli with concomitant regulation of immune system and cellular processing, while under-represented terms associated with locomotion, cell migration, secretion and localization (Fig. S6, Data S3–S4). Together, the DEG and gene-ontology enrichment analyses suggest that ARM are potentially stationary sensors of the environment and are primed to elicit an activated immune response.

To further validate these microglial subclusters, co-expression analysis was performed. Metacells obtained by combining statistically similar single nuclei (~50 nuclei per metacell) were used to create co-expression networks using a modified cell-specific network (CSNs) algorithm based on 477 significant DEGs obtained by ANOVA (Data S5) [29, 30]. Pairwise comparison of CSNs from homeostatic, motile, ARM and dystrophic microglia using multivariate data change point tests showed significant differences in CSNs between all subtypes (Table S5). To visualize these significant alterations, Sankey plots were constructed to show the flow of co-expression gene modules between co-expression matrices from each microglial subtype from homeostatic to motile, ARM and dystrophic (Fig. 2C). These results further confirmed the distinct gene expression patterns among the four microglial subclusters.

Microglial subcluster proportions were then determined in relation to pathologic classification and genotype. In *TREM2* wildtype cases, homeostatic microglia predominated in A−T− cases, while dystrophic microglia were exclusively seen, albeit in limited proportions, in A+T− and A+T+ cases (Fig. 2D). Interestingly, ARM were significantly increased in A+T− cases (77.5%) compared to A−T− cases (7.8%; Fisher’s exact test, OR 40.53, 95% CI 23.58 to 73.71, *p*-*value* <0.0001). Moreover, ARM were significantly reduced A+T+ cases (25.7%) compared to A+T− cases (Fisher’s exact test, *p-value* < 0.0001, OR 0.101, 95% CI 0.082 to 0.124) (Fig. 2D). Conversely, motile microglia were the major population in A+T+ cases, suggesting that the presence of neurofibrillary degeneration is associated with a shift in microglial response. To further analyze *TREM2* WT cases, microglial subcluster proportions were also stratified by *APOE* genotype and matched for pathology (Fig. 2E). ARM cells composed 61.2% of the microglial population in *APOE* E3/E3 cases and were significantly reduced to 45.5% in *APOE* E3/E4 cases (Fisher’s exact test, OR 0.528, 95% CI 0.435 to 0.641, *p-value* <0.0001), suggesting that *APOE* risk genotypes are associated with a reduced proportion of ARM microglia. Similarly, *TREM2* R47H cases, irrespective of pathologic classification, exhibited a relative paucity of ARM cells (15.0%) compared to *TREM2* WT A+T+ cases (Fisher’s exact test, OR 0.509, 95% CI 0.381 to 0.676, *p-value* <0.0001,) (Fig. 2F). Overall, microglial subcluster proportions appear to shift in response to amyloid and tau pathologies and are modified by genotype, most notably a reduction of ARM in cases with *APOE* E4 and *TREM2* R47H risk genotypes.

### Branched microglial transcriptional trajectory

**Fig. 3.**
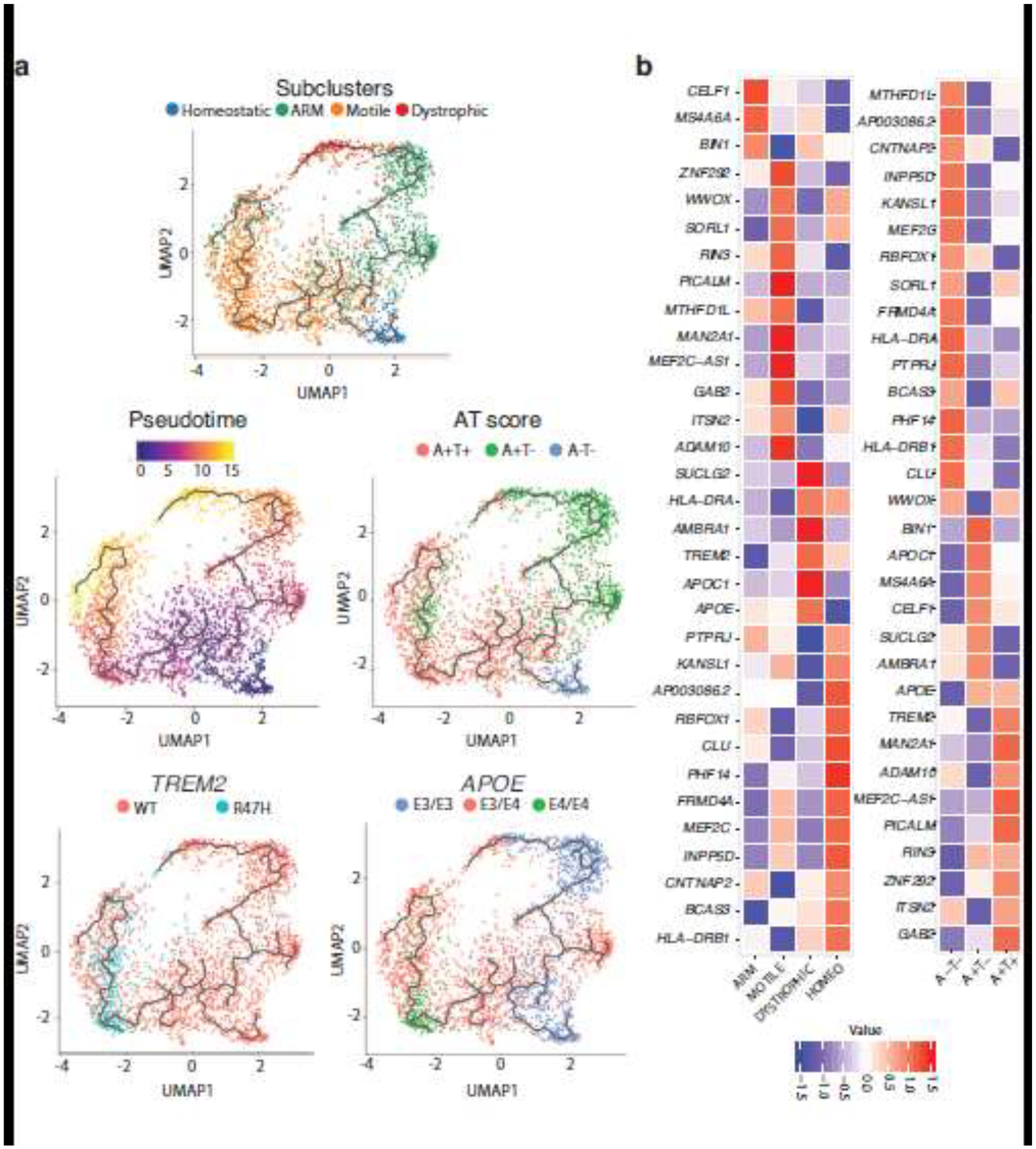
(a) Uniform Manifold Approximation and Projection (UMAP) visualization of the microglia cell trajectory colored by subclusters, estimated pseudotime, AT score, *TREM2* R47H and *APOE* genotype. (b) Heatmap of putative AD risk genes across microglia subpopulations (left) and AT score (right).

To evaluate whether the clusters show discrete transcriptional fates, trajectory reconstruction and pseudotime analyses were performed using Monocle 3 [32, 41, 42]. The microglial cells were aligned to a highly branched trajectory, with the main branch emanating from the homeostatic cluster and diverging into either motile microglia or ARM (Fig. 3A). Dystrophic microglia were found as an end state to the ARM (Fig. 3A). The nodal point and divergence of transcriptional trajectories between microglial subtypes appeared to be related to underlying neuropathologic classification and genotype as the motile microglia trajectory was associated with A+T+ pathology, *TREM2* R47H, and *APOE* E4. In contrast, the ARM/Dystrophic trajectory was associated with A+T− pathology, wildtype *TREM2*, and *APOE* E3. Thus, human microglia may respond to different underlying AD neuropathologies in a complex and non-linear fashion, somewhat different from single-cell RNA sequencing analyses performed in AD mouse models [14]. To validate these findings, a separate trajectory analysis using partition-based graph abstraction (PAGA) [43] was performed, which showed similar branched trajectories and associations (Fig. S7).

### Putative AD risk gene expression in microglial subpopulations

To explore whether expression of AD risk variants were associated with microglial phenotypes, expression of putative AD risk genes identified from genome-wide association studies were examined when grouping microglia by subcluster or pathology (Fig. 3B) [6, 10, 34]. Putative AD risk genes were upregulated in a discrete, often non-overlapping fashion by subcluster (Fig. 3B, left) and by pathology (Fig. 3B, right). ARM demonstrated the greatest-fold changes for risk genes *BIN1*, *CELF1*, and *MS4A6A*, wherein the MS4A gene cluster was recently shown to be a key genetic modulator of soluble TREM2 in the CSF [44].

### Reduced ARM response in APOE E4 and TREM2 R47H cases

**Fig.4.**
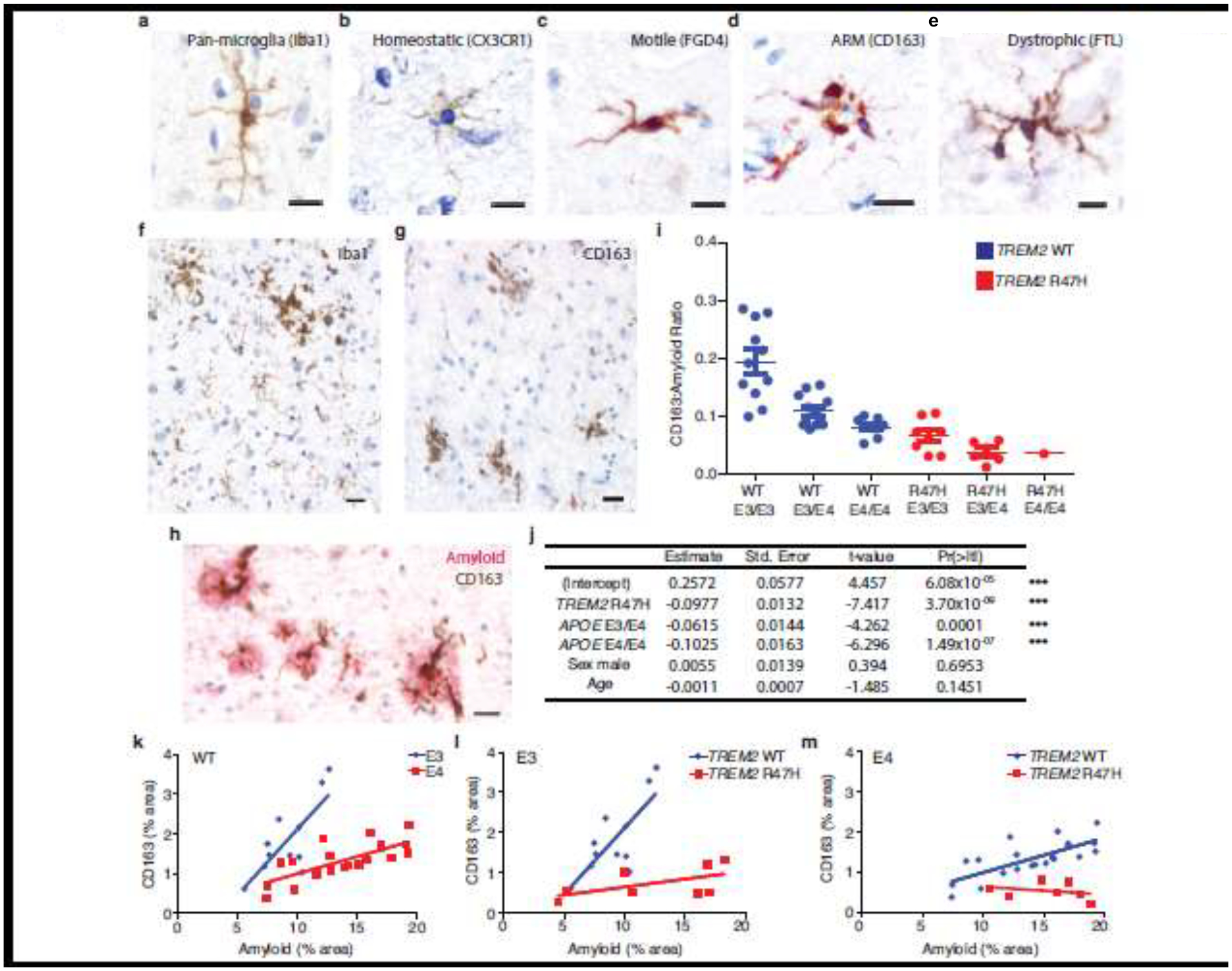
CD163+ ARM are associated with amyloid plaques and show decreased expression with *TREM2* R47H and *APOE* E4 allele. (a) Pan-microglial marker Iba1 stains microglia with a small cell body and highly ramified, branched processes in an adult brain without amyloid or tau pathology, and (b) homeostatic marker CX3CR1 highlights similar features in microglia, although astrocytes were also stained. In AD brains, (c) motile marker FGD4 highlights microglia with bipolar processes and hypertrophic cell bodies, (d) ARM marker CD163 shows clustered, amoeboid microglia, and (e) FTL highlights dystrophic microglia with hypertrophic cell bodies and beaded processes. (f) Iba1 staining shows clustered, amoeboid microglia and intervening evenly-dispersed microglia in neocortex of AD brain, while (g) CD163 highlights only clustered microglia. (h) CD163+ ARM (*brown*) co-localize with beta-amyloid plaques (*pink*) in AD brain. (i) CD163:amyloid ratios are decreased in cases with the *TREM2* R47H variant and *APOE* E4 allele. (j) Multiple linear regression model shows a significantly decreased CD163:amyloid ratio in (k) *TREM2* WT cases with the *APOE* E4 allele versus E3/E3. When matched for *APOE* allele, cases with the *TREM2* R47H variant showed a consistently decreased ARM:amyloid ratio in (l) *APOE* E3/E3 cases and (m) *APOE* E3/E4 cases. ***p < 0.001. Images (a-g) white balanced; scale bar, 10 μm.

Because AD risk genes *APOE* and *TREM2* have been suggested to attenuate microglial responses to pathology, the effects of *APOE* and *TREM2* on microglial phenotype, specifically ARM, were examined histopathologically in an expanded cohort of cases of neuropathologically defined AD versus non-AD tissues (Table S1) [14, 21, 22, 45, 46]. Immunohistochemical markers for each microglial subpopulation were explored using a list of statistically significant (adjusted p-value < 0.05) DEGs generated for each subcluster (Fig. S6). The known homeostatic microglial gene *CX3CR1* showed a statistically significant (adjusted *p-value* 0.001) 1.8 log2-fold change over ARM. CX3CR1 immunohistochemistry stained highly branched, ramified homeostatic microglia, similar to microglia from neurologically normal cases stained for the pan-microglial marker, Iba1 (Fig. 4A–B). For motile microglia, *FGD4* showed a 1.1 log2-fold change over homeostatic microglia (adjusted *p-value* 0.04) and stained microglia with amoeboid to uni- and bi-polar processes and larger cell bodies (Fig. 4C) in AD human brain tissue. The scavenger receptor gene *CD163* demonstrated a 5.1 log2-fold change over homeostatic microglia (adjusted *p-value* 2.48 × 10–7) in ARM cells versus homeostatic microglia, and IHC staining for CD163 showed clustered, amoeboid appearing microglia in AD human brain tissues (Fig. 4D). Lastly, the ferritin-light chain gene *FTL* showed a 3.9 log2-fold change in dystrophic microglia over homeostatic microglia (adjusted *p-value* 2.82 × 10–50). Immunohistochemistry in AD human brain tissues for ferritin-light chain demonstrated dystrophic microglia with a hypertrophied cell body and beaded processes as previously described [38] (Fig. 4E). Importantly, these four microglial markers appeared to label distinct, largely non-overlapping populations of microglia. Moreover, in normal (A−T−) human brain tissue, only very rare FGD4 positive cells were seen (~2–3 per case), and CD163-postive staining was limited to perivascular macrophages and negative in microglia (Fig. S8).

In human AD brain tissues, CD163 was a specific marker for ARM when compared to the pan-microglial marker Iba-1 (Fig. 4F–4G). CD163-positive ARM were seen exclusively associated with beta-amyloid plaques in contrast with homeostatic, motile, and dystrophic microglia, which were typically not associated with beta-amyloid plaques (Fig. 4H). Mice lacking *Trem2* show a limited response to beta-amyloid plaques [14]. Moreover, *Apoe* deletion has been suggested to reduce microglial reactivity to amyloid [21, 22]. Thus, we tested whether *TREM2* R47H and *APOE* E4 genotypes were associated with a reduction of a specific microglial subpopulation in AD human tissues. 48 AD cases with varying *TREM2* and *APOE* genotypes (Table S1) were immunostained for CX3CR1, FGD4, FTL, CD163, amyloid, and PU.1 (Table S6). Total microglia remained similar across cases, although sex-dependent differences in total microglia numbers were seen (Fig. S9). While markers for homeostatic and motile microglia did not reveal appreciable differences between cases, CD163 showed marked differences between genetic subgroups and were further analyzed using quantitative analysis of digitized histologic images. To measure the amount of ARM per plaque, a ratio of the percent area occupied by ARM versus percent area occupied by beta-amyloid was calculated for each case. ARM:amyloid ratios in *TREM2* R47H cases were significantly reduced compared to *TREM2* WT cases (Fig. 4I–4J), suggesting that the *TREM2* R47H genotype was associated with an impaired microglia response to amyloid. Moreover, *APOE* genotype manifested the same effect as there was a stepwise reduction in ARM per plaque with the addition of each *APOE* E4 allele (Fig. 4I–4J).

To visualize this relationship between ARM and amyloid, ARM (% area) was plotted as a function of amyloid (% area) (Fig. 4K–4M). A decreased amount of ARM to amyloid burden was seen in *APOE* E4 cases versus *APOE* E3 suggesting that *APOE* E4 attenuates the ARM response to amyloid (Fig. 4K). Similarly, there was a mitigated ARM response in *TREM2* R47H versus WT cases when controlled for *APOE* genotype, whether *APOE* E3 (Fig. 4L) or *APOE* E4 (Fig. 4M). These data suggest that both *APOE* E4 and *TREM2* R47H variants are associated with a dampened ARM response to beta-amyloid plaques, raising the possibility that *APOE* and *TREM2* risk genotypes may confer risk for AD by down modulation of the ARM response.

## Discussion

The neuroimmune landscape in AD is complex, with mounting evidence demonstrating how glial cells, particularly microglia, play a key role in disease [47, 48]. Whether the microglial response is harmful, helpful, or both during disease progression remains unclear. Using an unbiased snRNA-seq approach and a novel bioinformatics pipeline DESC, which utilizes a machine-learning algorithm to perform iterative clustering, to study human brain tissue, we show distinct cell populations within the human brain. After subclustering the microglia, four microglial populations emerged with distinctive transcriptional, pathologic, and genotypic profiles, suggesting heterogeneity in microglial subpopulations. Among these subpopulations was ARM, an amyloid responsive microglial subpopulation expressing CD163. Through differential expression analysis, co-expression module analysis, and trajectory reconstruction techniques, ARM was found to be a branch of homeostatic microglia, distinct from motile microglia, another reactive subpopulation. Moreover, the ARM response was reduced in human brain tissues with *APOE* E4 and *TREM2* R47H variants, demonstrating that AD risk genes appear to limit the CD163-positive ARM population in human disease.

Microglial heterogeneity has long been documented in the literature and has been characterized on morphologic, immunohistochemical, and functional levels [49–51]. To date, few studies utilizing single-cell or single-nucleus RNA sequencing of human tissue have shown the granularity of the microglial response in AD, particularly to beta-amyloid deposition. Here, we demonstrate the capability of resolving distinct CNS populations in postmortem human brain which we validate neuropathologically with a well-characterized series of human brain autopsy cases, allowing for a better understanding of microglia population dynamics in AD. For example, snRNA-seq of human tissues has demonstrated sexually dimorphic cellular responses in AD [22, 52–54]. Due to the known sexual dimorphism of microglia, we chose to focus our snRNAseq analysis on male cases. However, we do corroborate that there is a sexually dimorphic microglial response in AD in our larger immunohistochemistry validation cohort which included both male and female cases (Fig.S8). Similar to another recent snRNA-seq study in human AD tissue, we show loss of neurons with concomitant gliosis in AD brain compared to normal cases[55]. However, we did not observe the same *IRF8*-driven reactive phenotype or a generalized downregulation of metal-ion homeostasis. Rather by utilizing DESC, we were able to obtain a finer resolution of microglia subpopulations consisting of four key microglia subpopulations emerged: homeostatic, motile, ARM, and dystrophic. DESC is an unsupervised deep-embedding algorithm for single-cell or single-nucleus clustering. By allowing for machine-learning based iterative clustering and providing a probability for each cell or nucleus determining the confidence of cluster assignment, it provides a way to remove technical artifacts like batch effect and also allows for high fidelity clustering [37].

The homeostatic subpopulation demonstrated *CX3CR1* as a statistically-significant upregulated differentially expressed gene, while other homeostatic markers such as *TMEM119* and *P2RY12* were not significant in our study, perhaps due to the sparsity of data inherent to snRNA-seq. Potential markers for the other microglial subpopulations were identified based on DEGs (*FGD4*, *FTL*, and *CD163* for motile, dystrophic, and ARM, respectively) and examined in an expanded autopsy cohort. ARM are characterized by their specific staining for CD163, a transmembrane scavenger receptor that is part of the scavenger receptor cysteine-rich (SRCR) domain family [56, 57]. While CD163 is mostly known as a scavenger receptor for hemoglobin-haptoglobin complexes and aids in receptor-mediated endocytosis after hemorrhage, it has a wide variety of immunoregulatory functions including, but not limited to, an innate immune sensor of bacteria, receptor for TNF-like weak inducer of apoptosis, and an attachment point for a number of viruses [58,59]. Moreover, its soluble form may play a role in opsonizing bacteria to aid in phagocytosis [60]. While in neurologically normal postmortem human brain tissue, CD163 staining is limited to perivascular macrophages as seen in our results and observed in other studies, it has been shown to be upregulated in microglia in disease states such as Alzheimer’s disease, Parkinson’s disease, multiple sclerosis, HIV encephalitis, SIV encephalitis, and traumatic brain injury [61–63]. In human AD tissue, CD163-positive microglia have been shown in close apposition to beta-amyloid plaques, particularly in neuritic plaques, and demonstrated increased expression of CD68, a lysosomal marker, suggesting increased phagocytosis [62]. Though the mechanism remains unclear, ARM may act as a defense against beta-amyloid accumulation through barrier formation and increased phagocytosis.

A heterogenous microglial response to AD neuropathologic inclusions has been seen in mice. Reactive mouse microglial populations, termed variably disease-associated microglia (DAM), microglial neurodegenerative phenotype (MGnD), or activated response microglia, are conceptually similar to the ARM described here in that they all appear to be associated with beta-amyloid plaques [14, 22, 45]. However, when comparing differentially expressed genes in these microglia subtypes and ARM, most genes did not overlap between subclusters with only *SPP1* was shared between all subclusters and ARM (Fig. S10). Overall, ARM DEGs overlapped the most with MGnD, but still only sharing genes *APOE*, *SPP1*, and *TLR2* (Fig. S10). Osteopontin, the protein encoded by *SPP1*, is a potential marker for activated microglia and macrophages [64]. Moreover, in various rodent models secreted osteopontin acted as an opsonin for apoptotic cellular debris, ultimately facilitating increased phagocytosis [65].

Despite these differences between human and mouse microglial responses, activated mouse microglia and human ARM do share some similarities including the increased expression of APOE. Microglia have been demonstrated to express APOE protein in both mouse and human microglia, particularly in plaque-associated microglia [66–72]. In contrast, we observed TREM2 expression to be relatively decreased in ARM [73]. Although TREM2 protein expression is increased in AD, TREM2 protein levels do not correlate with amyloid plaque accumulation [73]. Rather, increased expression of TREM2 is frequently seen in microglia not associated with amyloid plaques but in close relation to tau positive dystrophic neurites [73]. Notably, many microglial genes including *APOE*, *TREM2*, and even the homeostatic microglial marker, *CX3CR1*, appear to demonstrate graded expression changes upon activation. In contrast, CD163 appeared to be remarkably binary in terms of its expression, being essentially absent in microglia from neurologically normal controls and in non-plaque associated microglia.

Several recent studies have examined the relationship between *Apoe*, *Trem2*, and microglia, predominantly in murine models [14, 21, 22, 45, 52, 55]. scRNA-seq analysis of *Apoe* knockout in AD amyloid mouse models have shown reduced numbers of activated response microglia, suggesting that *Apoe* attenuates the microglial response to beta-amyloid in mice [22]. A similar reduction was seen on a histological level in fibrillar plaque-associated microgliosis in *Apoe*-deficient mice with AD amyloid pathology [21]. Correspondingly, by identifying a specific marker of ARM, our study found that the anti-amyloid microglial response was reduced according to *APOE* E4 in a step-wise fashion: cases with either one or two copies of *APOE* E4 demonstrated a reduced response to beta-amyloid when compared to *APOE* E3. Thus, the human ARM response, whilst demonstrating some transcriptomic differences relative to mouse activated response microglia, appears to be similarly influenced by *APOE* in the setting of beta-amyloid deposition.

snRNA-seq of AD human tissue has also shown an overall decreased expression of genes related to microglial activation in *TREM2* R47H cases without identifying a specific microglial subpopulation that is related to AD risk [55]. We further refine our understanding of the cellular responses in human AD tissues by showing that *TREM*2 R47H is associated with a reduction in a specific subpopulation of ARM rather than a generalized loss of microglial reactivity [55]. Moreover, using co-expression module analysis and trajectory reconstruction, the relationships between the human microglial subpopulations, particularly reactive populations, were found to be branched, rather than linear. This differs from the trajectory seen in murine DAM populations where stages of microglial activation in 5xFAD mice were sequential, with the final stage dependent upon *TREM2* activation (i.e. stage 2 DAM). We found that the *TREM2* R47H AD risk variants are associated with a shift in microglial phenotype from ARM to motile, and that the ARM and motile microglia appear to represent distinct activation phenotypes emanating from homeostatic microglia. While ARM are conceptually similar to stage 2 DAM, the branched and complex subpopulation trajectory of human microglia suggests that the innate immune response is complicated and heterogeneous, related to underlying pathology and genetics.

A limitation of this study is its correlative nature where we assume that microglia are responding to amyloid due to their spatial proximity to each other. Overall, the ARM response appears as one facet of the microglial response in AD and is closely associated with beta-amyloid plaques. Furthermore, the ARM response is reduced in the setting of sporadic AD genetic risk variants such as *TREM2* R47H and *APOE* E4. While it is plausible that the ARM response to beta-amyloid plaques is protective, the precise mechanism remains unclear. However, the reduction of beta-amyloid plaque-associated microglia resulted in increased neuronal dystrophy, suggesting that these plaque-associated microglia may be protective against downstream pathologic processes [46, 74].

The beta-amyloid cascade hypothesis faces scrutiny due to the failure of a few beta-amyloid targeted therapeutics [75, 76]. However, it remains important to continue to examine how beta-amyloid deposition affects cellular phenotypes, particularly in the context of neuroinflammation. This study suggests that the disarming of an activated microglial subpopulation, ARM, may be associated with enhanced risk for AD, indicating that potential immunomodulatory therapeutics may need to discern between protective and deleterious effects of the activated microglial response.

## Supplementary Material

Data S3

Data S1

Data S4

Suppl Tables and Figures

Data S5

Data S2
