# Supplementary material for: APOE and TREM2 regulate amyloid responsive microglia in Alzheimer’s disease
Source: Acta Neuropathol. Author manuscript; Available in PMC 2021 Oct 1. (PMC7520051; doi:10.1007/s00401-020-02200-3)
Supplement: Suppl Tables and Figures [file NIHMS1623526-supplement-Suppl_Tables_and_Figures.pdf]

Title: *APOE* and *TREM2* regulate amyloid responsive microglia in Alzheimer's disease

Authors: Aivi T. Nguyen[1,7]†, Kui Wang[2,3]†, Gang Hu[2,4]†, Xuran Wang[5], Zhen Miao[6], Joshua A. Azevedo[1,7], EunRan Suh[7], Vivianna M. Van Deerlin[7], David Choi[8], Kathryn Roeder[5,9], Mingyao Li[2*], Edward B. Lee[1,7*]

Affiliations:

[1]Translational Neuropathology Research Laboratory, Department of Pathology and Laboratory Medicine, Perelman School of Medicine at the University of Pennsylvania, PA, USA.

[2]Department of Biostatistics, Epidemiology and Informatics, Perelman School of Medicine at the University of Pennsylvania, PA, USA.

[3]Department of Information Theory and Data Science, School of Mathematical Sciences and LPMC, Nankai University, Tianjin, China.

[4]School of Statistics and Data Science, Key Laboratory for Medical Data Analysis and Statistical Research of Tianjin, Nankai University, Tianjin, China.

[5]Department of Statistics and Data Science, Carnegie Mellon University, PA, USA.

[6]Graduate Group in Genomics and Computational Biology, Perelman School of Medicine at the University of Pennsylvania, PA, USA.

[7]Department of Pathology and Laboratory Medicine, Perelman School of Medicine at the University of Pennsylvania, PA, USA

[8]Heinz College of Public Policy and Information Systems, Carnegie Mellon University, PA, USA

[9]Computational Biology Department, Carnegie Mellon University, PA, USA

†Equal contributions

*Co-corresponding authors

Mingyao Li
Department of Biostatistics, Epidemiology, and Informatics
213 Blockley Hall, 423 Guardian Drive
Philadelphia, PA 19104
(215) 746-3916
mingyao@pennmedicine.upenn.edu

Edward B. Lee
613A Stellar Chance Laboratories
422 Curie Blvd
Philadelphia, PA 19104
(215) 898-0908
edward.lee@pennmedicine.upenn.edu

This PDF file includes:

Figs. S1 to S10
Tables S1 to S6
Captions for Data S1 to S5

Other Supplementary Materials for this manuscript include the following:

Data S1 to S5

- **Data S1.** The DE gene lists of ARM, motile and dystrophic vs homeostatic.
- **Data S2.** The DE gene lists of for homeostatic, ARM, motile and dystrophic (one vs. rest).
- **Data S3.** GO enrichment analysis in Biological Processes ontology between ARM, motile and dystrophic vs homeostatic.
- **Data S4.** GO enrichment analysis in reactome pathways between ARM, motile, dystrophic and homeostatic (one vs. rest)
- **Data S5.** The DE gene list for the subclusters in homeostatic, ARM, motile, and dystrophic microglia using MetaCell data

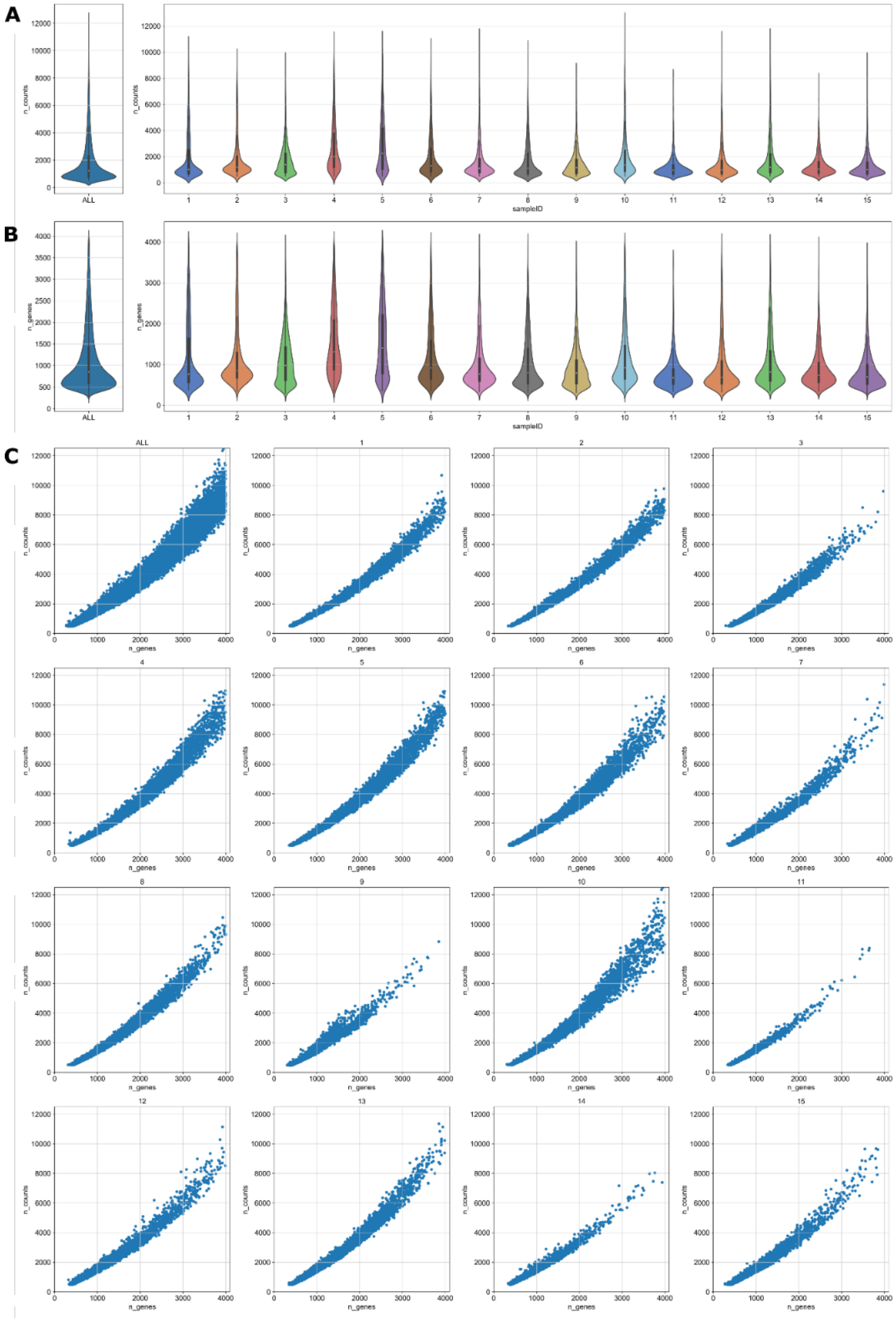

Fig. S1. Overview and quality control of the snRNA-seq data. (A) Violin plots of total UMI counts. Left is the violin plot of total UMI counts across all samples. The right panel shows violin plots of total UMI counts for each individual sample. (B) The violin plots of expressed number of genes. Left is the violin plot of the expressed number of genes across all samples. The right shows violin plots of the expressed number of genes for each individual sample. (C) Dot plots of expressed genes as a function of expression counts for all individual samples.

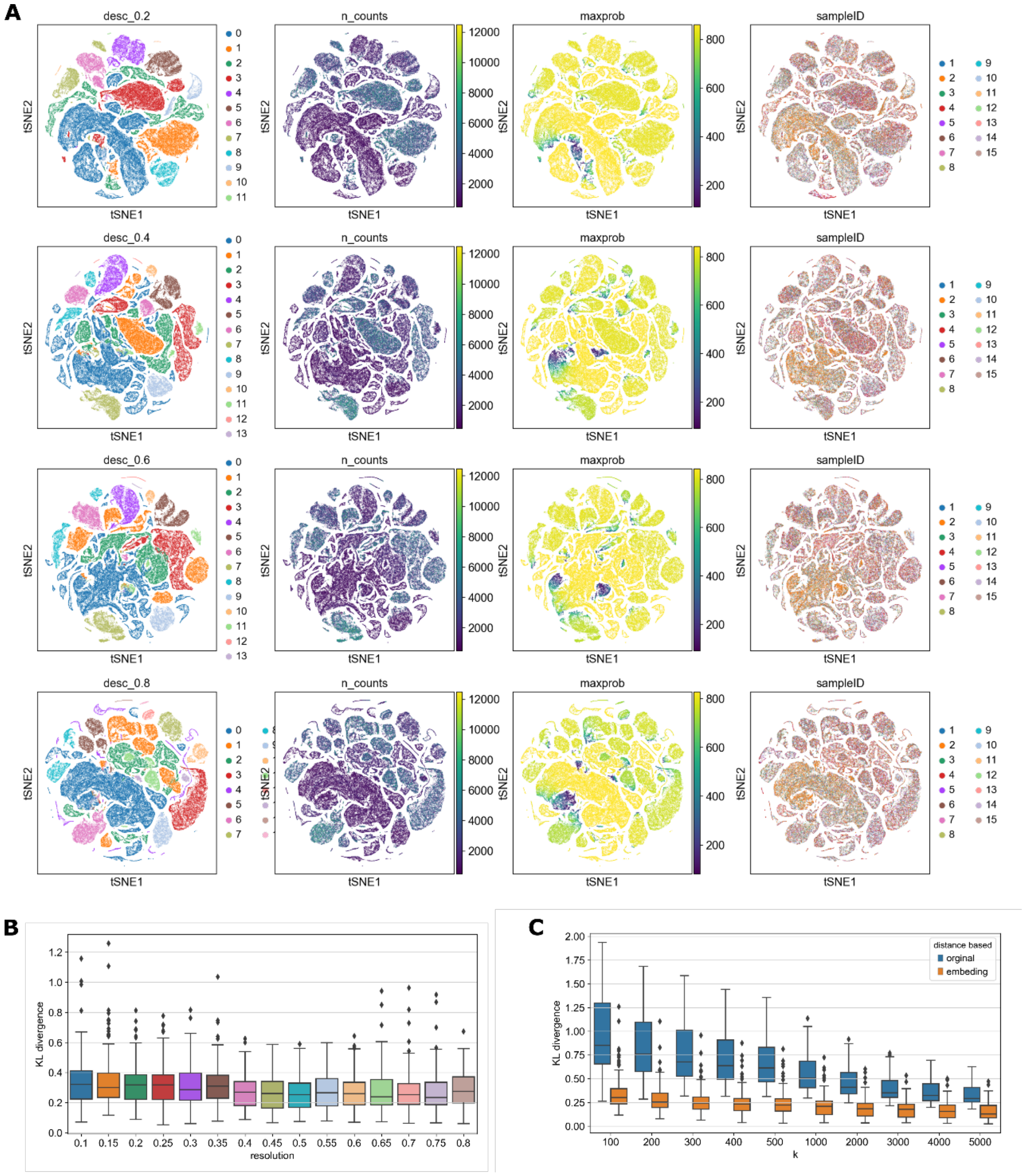

Fig. S2. Clustering results using different resolutions in DESC. (A) The clustering results from four resolutions 0.2, 0.4, 0.6, and 0.8 are shown as t-SNE plots. The first column demonstrates t-SNE plots colored by clusters. The second column is t-SNE plots colored by the total UMI counts. The third column is t-SNE plots colored by maximum probability for cluster assignment from DESC. The maximum probability was multiplied by 1000 (e.g., 800 means 0.8) for displaying purpose. The forth column is t-SNE plots colored by samples, which shows that the nuclei from different samples mixed well and the batch effect was removed effectively in clustering. (B) The boxplots of KL divergence based on embedding from DESC. (C) The

boxplots of KL divergence based on DESC embedding space (resolution was set at 0.15) and original expression space when varying the value of *K*.

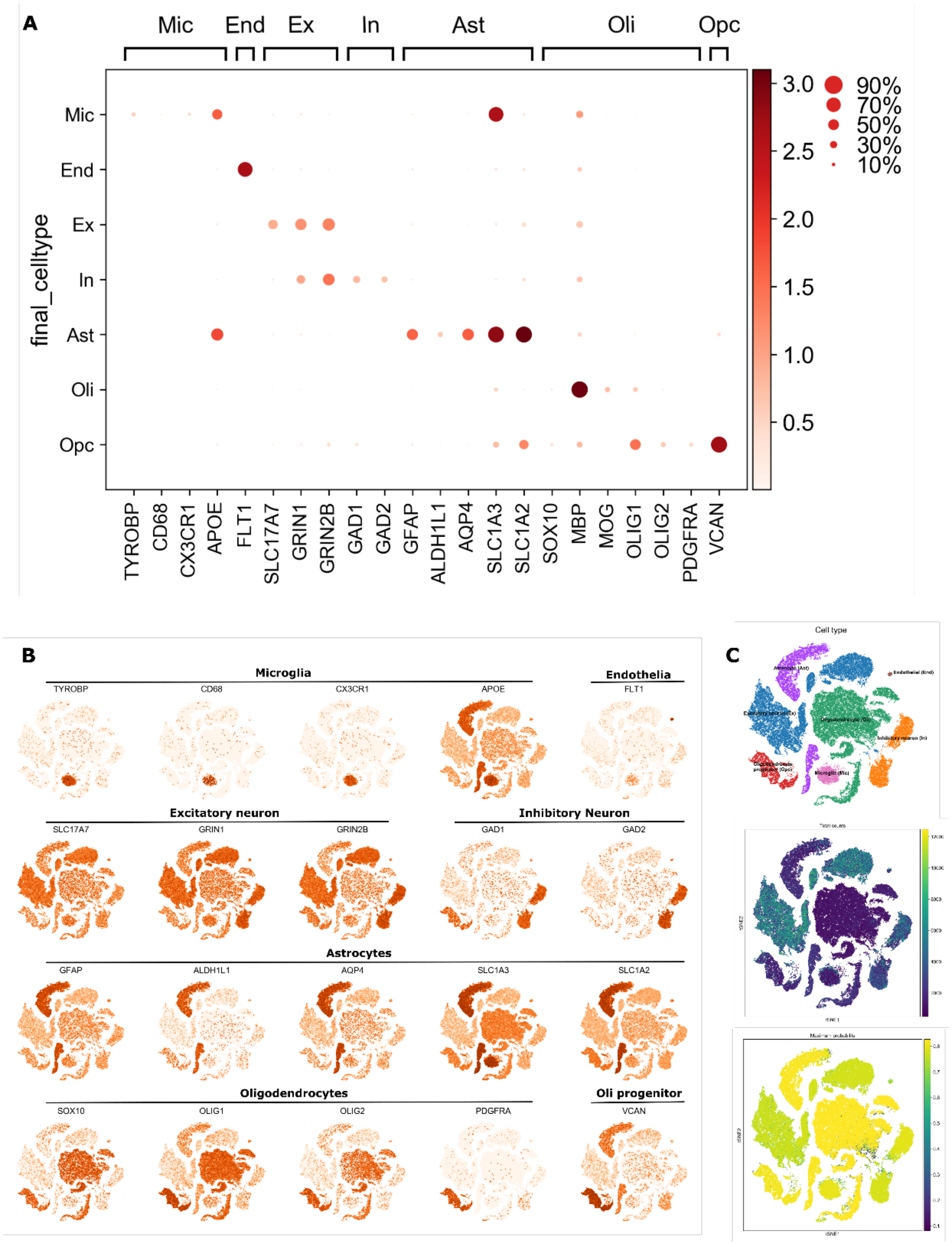

Fig. S3. Gene expression plots for marker genes of different cell types. (A) The dotplot of the marker genes for each annotated cell type. (B) The t-SNE plots colored by known marker genes. (C) The t-SNE plots colored by cell type, total UMI counts and maximum probability for cluster assignment.

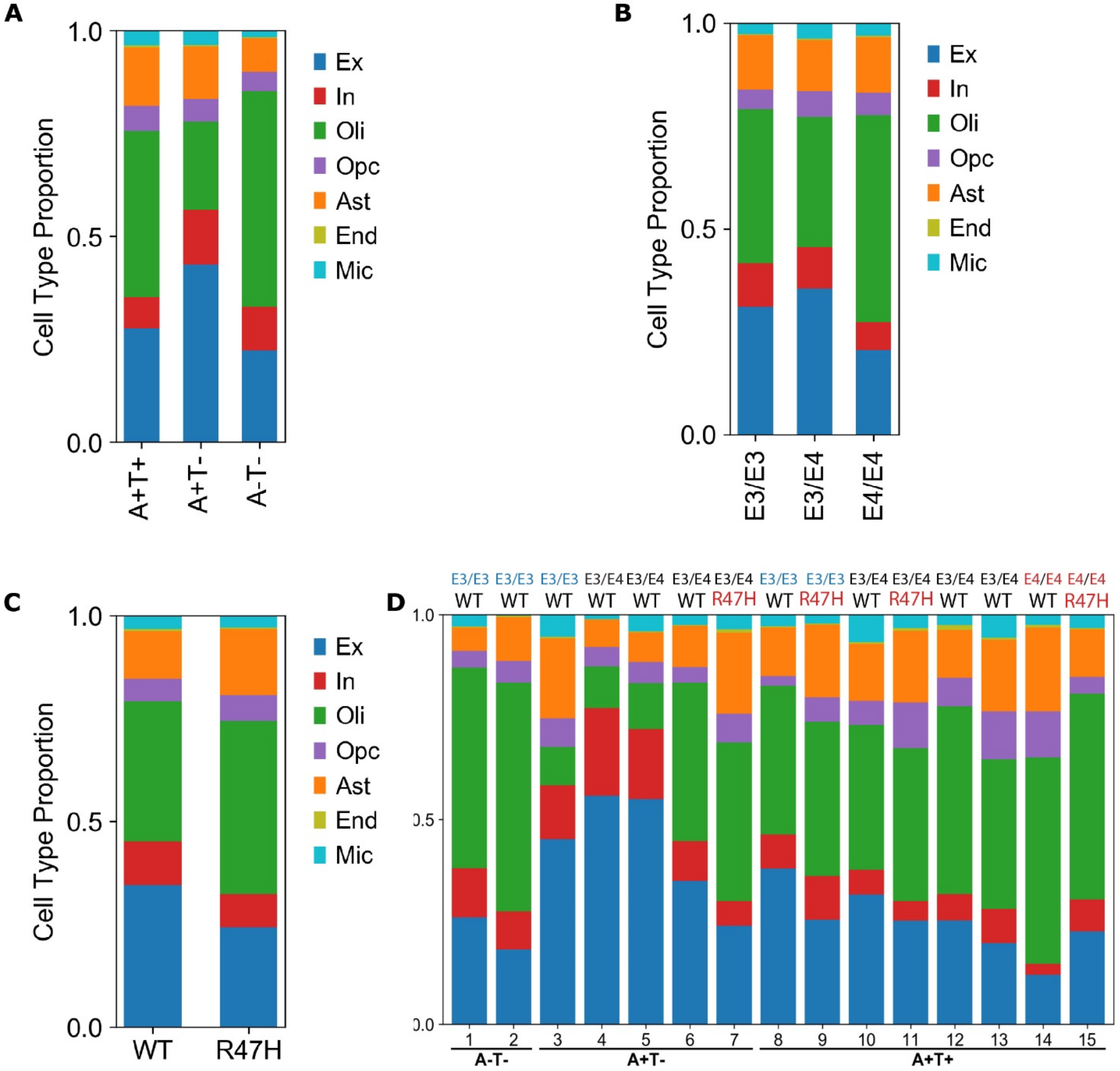

Fig. S4. Cell type proportions across samples with different pathology. (A) Cell type proportions in samples with different AT scores. (B) Cell type proportions across different samples with *APOE* genotypes. (C) Cell type proportions in samples by *TREM2* allele, samples with the wild type (WT) *TREM2* allele, and samples with the R47H mutation for *TREM2*. (D) Cell type proportion in each individual sample.

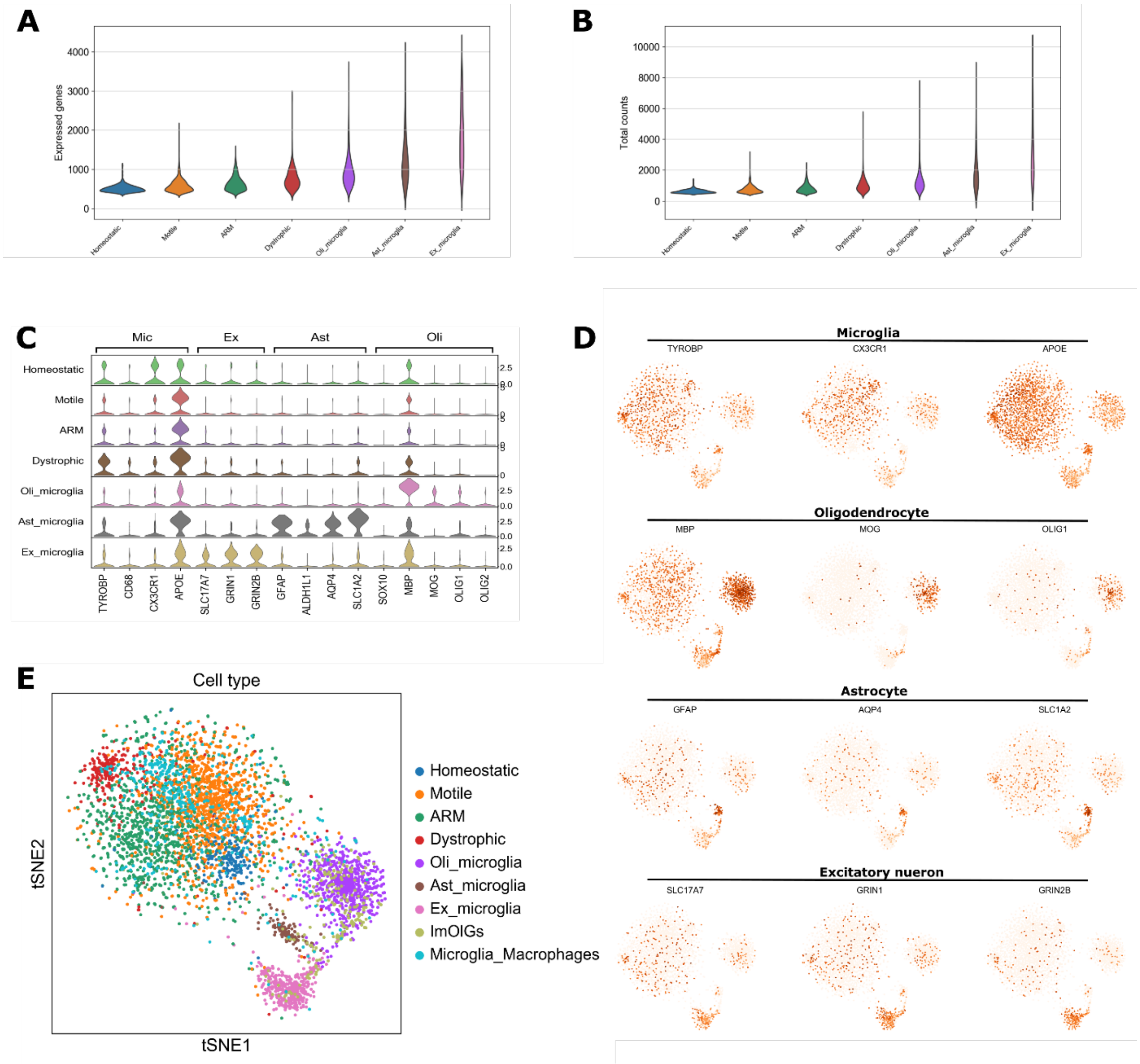

Fig. S5. Contamination analysis for initially identified microglia. (A) The violin plot of the number of expressed genes in the 7 subpopulations. (B) The violin plot of total UMI counts in the seven subpopulations. (C) The violin plot of marker genes for microglia, excitatory neurons, astrocytes, and oligodendrocytes in the 7 subpopulations. (D) The t-SNE plots colored by different marker genes (microglia, oligodendroctyes, astrocytes and excitatory neurons). (E) The t-SNE plot for the combined analysis with the Jakel *et al.* dataset to include ImOlGs and

Microglia_Macrophages. ImOlGs were mixed with Oli_microglia and Microglia_Macrophages were mixed with the main four subpopulations for microglia.

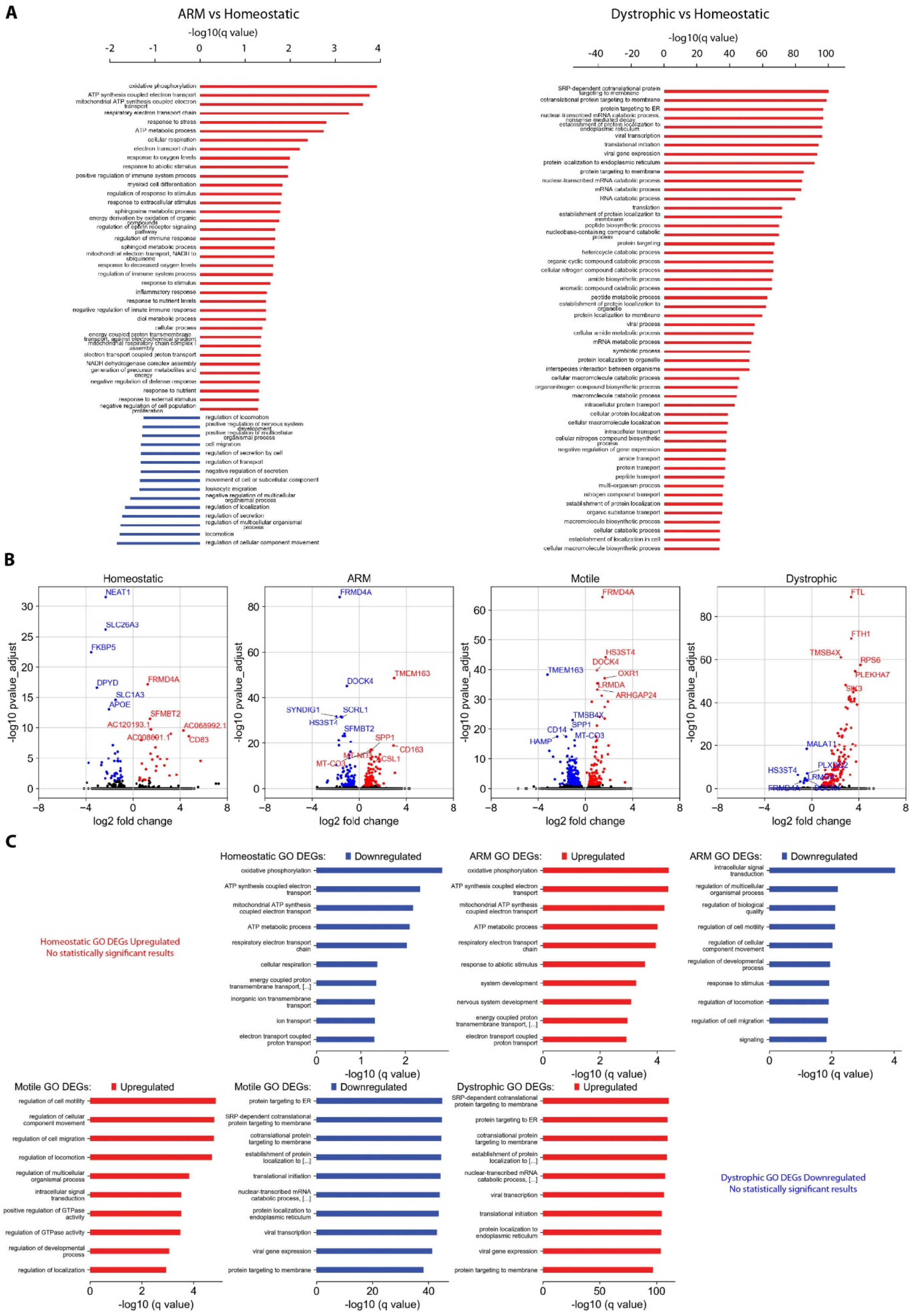
A
ARM vs Homeostatic
-log10(q value)
oxidative phosphorylation
ATP synthesis coupled electron transport
mitochondrial ATP synthesis coupled electron transport
respiratory electron transport chain
response to stress
ATP metabolic process
cellular respiration
electron transport chain
response to oxygen levels
response to abiotic stimulus
positive regulation of immune system process
myeloid cell differentiation
regulation of response to stimulus
response to extracellular stimulus
sphingosine metabolic process
energy derivation by oxidation of organic compounds
regulation of ephrin receptor signaling pathway
regulation of immune response
sphingoid metabolic process
mitochondrial electron transport, NADH to ubiquinone
response to decreased oxygen levels
regulation of immune system process
response to stimulus
inflammatory response
response to nutrient levels
negative regulation of innate immune response
diol metabolic process
cellular process
energy coupled proton transmembrane transport, against electrochemical gradient
mitochondrial respiratory chain complex I assembly
electron transport coupled proton transport
NADH dehydrogenase complex assembly
generation of precursor metabolites and energy
negative regulation of defense response
response to nutrient
response to external stimulus
negative regulation of cell population proliferation
regulation of locomotion
positive regulation of nervous system development
positive regulation of multicellular organismal process
cell migration
regulation of secretion by cell
regulation of transport
negative regulation of secretion
movement of cell or subcellular component
leukocyte migration
negative regulation of multicellular organismal process
regulation of localization
regulation of secretion
regulation of multicellular organismal process
locomotion
regulation of cellular component movement
Dystrophic vs Homeostatic
-log10(q value)
SRP-dependent cotranslational protein targeting to membrane
cotranslational protein targeting to membrane
protein targeting to ER
nuclear-transcribed mRNA catabolic process, nonsense-mediated decay
establishment of protein localization to endoplasmic reticulum
viral transcription
translational initiation
viral gene expression
protein localization to endoplasmic reticulum
protein targeting to membrane
nuclear-transcribed mRNA catabolic process
mRNA catabolic process
RNA catabolic process
translation
establishment of protein localization to membrane
peptide biosynthetic process
nucleobase-containing compound catabolic process
protein targeting
heterocycle catabolic process
organic cyclic compound catabolic process
cellular nitrogen compound catabolic process
amide biosynthetic process
aromatic compound catabolic process
peptide metabolic process
establishment of protein localization to organelle
protein localization to membrane
viral process
cellular amide metabolic process
mRNA metabolic process
symbiotic process
protein localization to organelle
interspecies interaction between organisms
cellular macromolecule catabolic process
organonitrogen compound biosynthetic process
macromolecule catabolic process
intracellular protein transport
cellular protein localization
cellular macromolecule localization
intracellular transport
cellular nitrogen compound biosynthetic process
negative regulation of gene expression
amide transport
protein transport
peptide transport
multi-organism process
nitrogen compound transport
establishment of protein localization
organic substance transport
macromolecule biosynthetic process
cellular catabolic process
establishment of localization in cell
cellular macromolecule biosynthetic process
B
Homeostatic
NEAT1
SLC26A3
FKBP5
DPYD
SLC1A3
APOE
FRMD4A
SFMBT2
AC120193.1
AC068992.1
AC008691.1
CD83
ARM
FRMD4A
DOCK4
TMEM163
SYNDIG1
SORL1
HS3ST4
SFMBT2
SPP1
CD163
MT-CO3
ACSL1
Motile
FRMD4A
HS3ST4
DOCK4
OXR1
TMEM163
LRMDA
ARHGAP24
TMSB4X
SPP1
CD14
HAMP
MT-CO3
Dystrophic
FTL
FTH1
TMSB4X
RPS6
PLEKHA7
SIK3
MALAT1
HS3ST4
FRMD4A
-log10 pvalue_adjust
log2 fold change
C
Homeostatic GO DEGs Upregulated
No statistically significant results
Homeostatic GO DEGs: Downregulated
oxidative phosphorylation
ATP synthesis coupled electron transport
mitochondrial ATP synthesis coupled electron transport
ATP metabolic process
respiratory electron transport chain
cellular respiration
energy coupled proton transmembrane transport, [...]
inorganic ion transmembrane transport
ion transport
electron transport coupled proton transport
ARM GO DEGs: Upregulated
oxidative phosphorylation
ATP synthesis coupled electron transport
mitochondrial ATP synthesis coupled electron transport
ATP metabolic process
respiratory electron transport chain
response to abiotic stimulus
system development
nervous system development
energy coupled proton transmembrane transport, [...]
electron transport coupled proton transport
ARM GO DEGs: Downregulated
intracellular signal transduction
regulation of multicellular organismal process
regulation of biological quality
regulation of cell motility
regulation of cellular component movement
regulation of developmental process
response to stimulus
regulation of locomotion
regulation of cell migration
signaling
Motile GO DEGs: Upregulated
regulation of cell motility
regulation of cellular component movement
regulation of cell migration
regulation of locomotion
regulation of multicellular organismal process
intracellular signal transduction
positive regulation of GTPase activity
regulation of GTPase activity
regulation of developmental process
regulation of localization
Motile GO DEGs: Downregulated
protein targeting to ER
SRP-dependent cotranslational protein targeting to membrane
cotranslational protein targeting to membrane
establishment of protein localization to [...]
translational initiation
nuclear-transcribed mRNA catabolic process, [...]
protein localization to endoplasmic reticulum
viral transcription
viral gene expression
protein targeting to membrane
Dystrophic GO DEGs: Upregulated
SRP-dependent cotranslational protein targeting to membrane
protein targeting to ER
cotranslational protein targeting to membrane
establishment of protein localization to [...]
nuclear-transcribed mRNA catabolic process, [...]
viral transcription
translational initiation
protein localization to endoplasmic reticulum
viral gene expression
protein targeting to membrane
Dystrophic GO DEGs Downregulated
No statistically significant results
-log10 (q value)

Fig. S6. Differential expression (DE) and GO Enrichment analysis. (A) The GO enrichment items based on the DE genes between ARM vs homeostatic (left) and dystrophic vs homeostatic (right). The items in red are based on upregulated DE genes and those in blue are based on downregulated DE genes. There is no significant GO enrichment items on the DE genes between motile vs homeostatic. (B) DE genes between each subpopulation versus other subpopulations combined. Genes colored by blue are down-regulated and genes colored by red are up-regulated. (C) GO enrichment analysis based on the DE genes (one subpopulation versus the rest of the subpopulations combined).

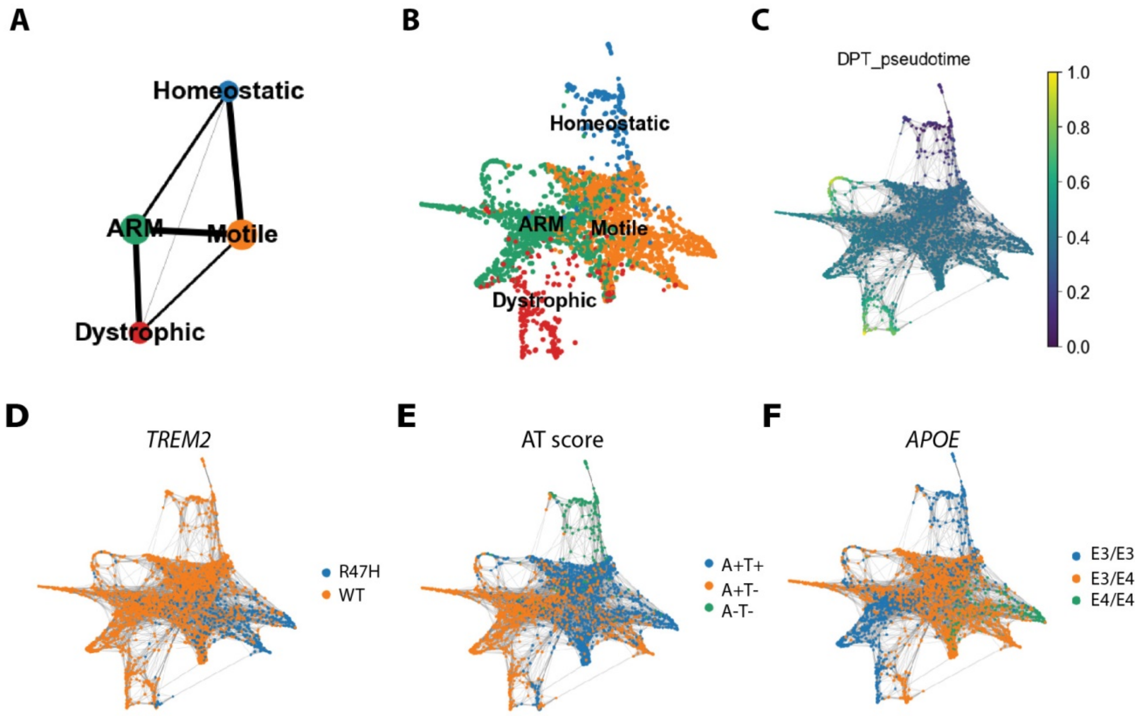

Fig. S7. Alternative analyses of microglial subclusters using partition-based graphical analysis (PAGA) and ForceAtlas2. (A) PAGA graph of microglial subclusters highlights the relationship between the 4 microglial subpopulations: homeostatic, motile, ARM, and dystrophic. The distance between nodes and weights of the connecting lines represent the connectivity between subclusters. (B) The ForceAtlas2 layout (Jacomy et al. 2014) of the microglial subclusters. ForceAtlas2, a force directed layout that recapitulates a physical system, allows interpretation of the subpopulations whereby nodes of each subpopulation repulse one another like charged particles, while the edges are meant to attract their respective nodes. This demonstrates how each subpopulation relates to one another and how they are interdependent. (C-F) The ForceAtlas2 plot with edges colored by (C) diffusion pseudotime, (D) *TREM2* genotype, (E) AT score and (F) *APOE* genotype.

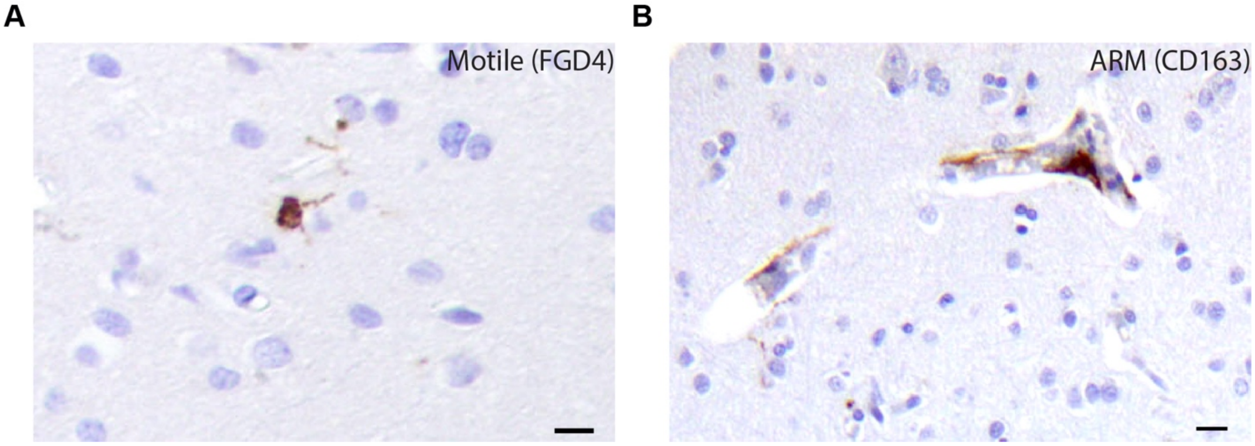

Fig. S8. Motile microglia and ARM staining in normal (A-T-) human brain tissue. Normal cases were stained for (A) FGD4, a marker for motile microglia, which showed very rare (~2-3) cells per case with a small cell body and thin ramified processes, reminiscent of microglia. (B) CD163, the ARM marker, highlighted perivascular macrophages and was negative in the parenchyma. Images white balanced. Scale bar, 10 µm.

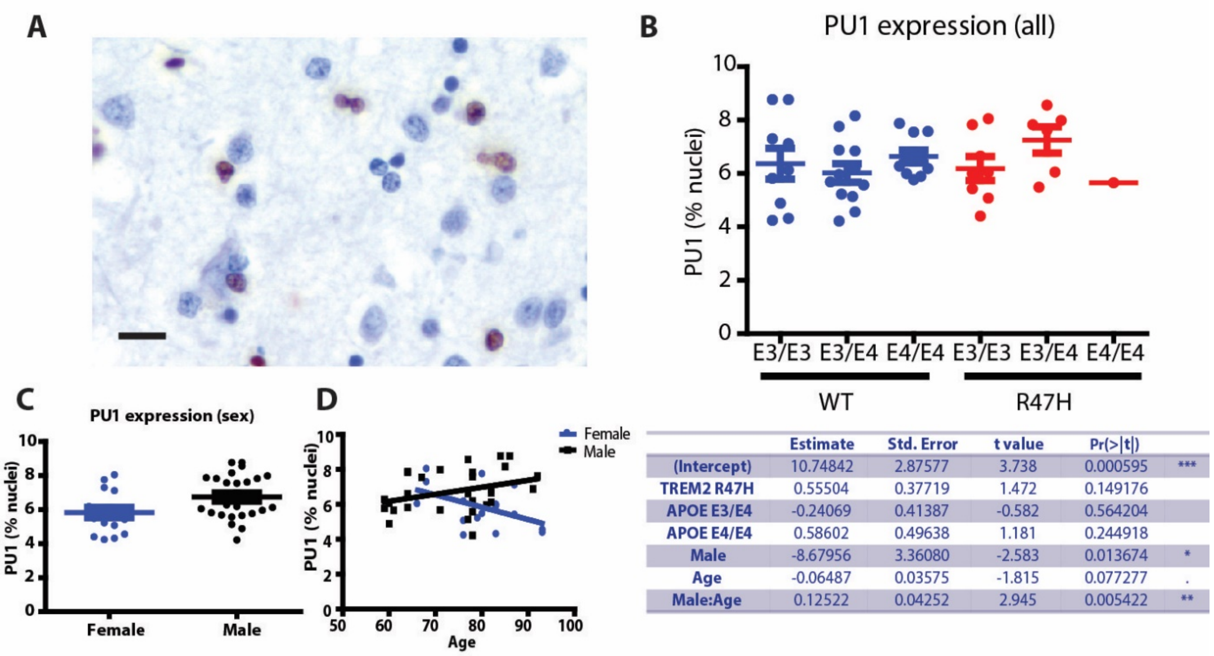

	Estimate	Std. Error	t value	Pr(>|t|)	
(Intercept)	10.74842	2.87577	3.738	0.000595	***
TREM2 R47H	0.55504	0.37719	1.472	0.149176	
APOE E3/E4	-0.24069	0.41387	-0.582	0.564204	
APOE E4/E4	0.58602	0.49638	1.181	0.244918	
Male	-8.67956	3.36080	-2.583	0.013674	*
Age	-0.06487	0.03575	-1.815	0.077277	.
Male:Age	0.12522	0.04252	2.945	0.005422	**

Fig. S9. Sexual dimorphism of PU.1 microglial nuclei across cases.
(A) IHC of PU.1 staining microglial nuclei in neocortex. Image white balanced. Scale bar, 10 µm. (B) A multiple linear regression model shows no statistical difference in PU.1 expression as a function of *TREM2* R47H variant or varying *APOE* genotypes. However, PU.1 expression decreases as a function of sex (*$p < 0.05$) and age (. $p < 0.10$). (C) PU.1 expression is decreased in females versus males, and decreases with age (D) in females.

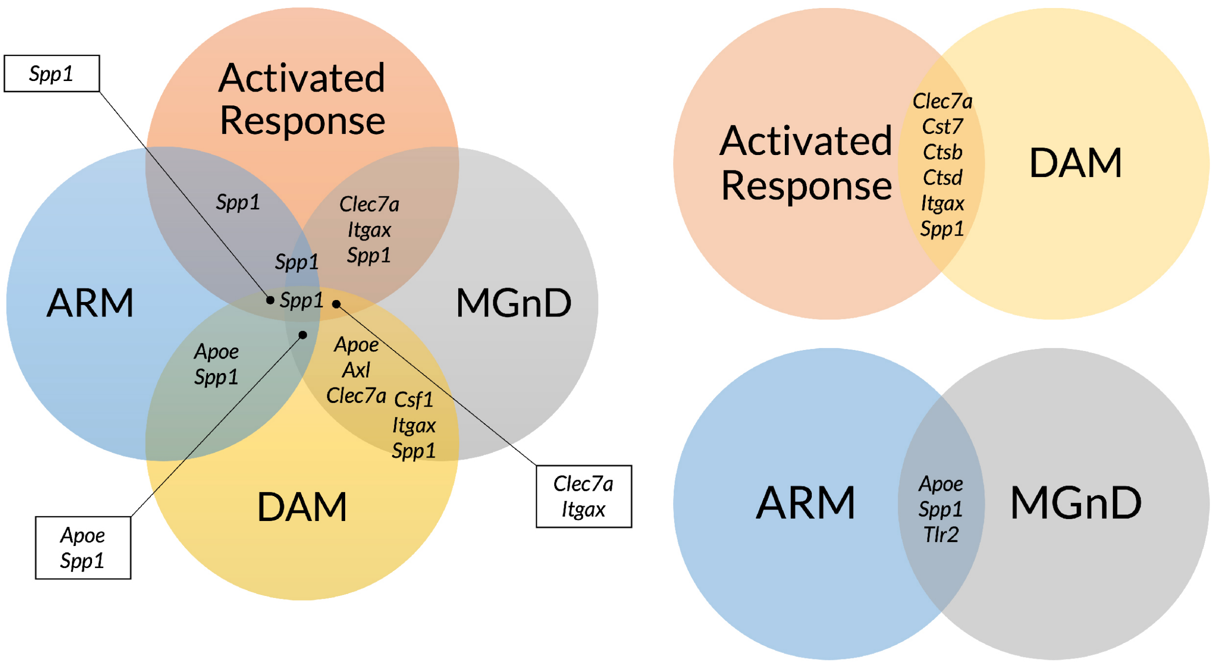

Fig. S10. Venn diagram of shared upregulated DEGs in activated response microglia (Krasemann et al. 2017), disease-associated microglia (DAM), including stage 1 and stage 2 DAM (Keren-Shaul et al. 2017), microglial neurodegenerative phenotype (MGnD) (Sala Frigerio et al. 2019), and amyloid responsive microglia (ARM).

Table S1. snRNA-seq sample demographics and pathology with expanded autopsy cohort characteristics

Sample Number	*APOE* Genotype	*TREM2 c.140G>A, p.Arg47His*	Sex	Age at death	Post-mortem Interval	Clinical Diagnosis(es)	Neuropathological Diagnosis	AT score	snRNA-seq	Immunohistochemistry
1	E3/E3	No	Male	70	36	Neurologically normal	Unremarkable adult brain	A-T-	x	
2	E3/E3	No	Male	59	17	Neurologically normal	Primary age-related tauopathy	A-T-	x	
3	E3/E3	No	Male	84	23	Neurologically normal	Low ADNC	A+T-	x	x
4	E3/E4	No	Male	57	14	Neurologically normal	Low ADNC, Cerebrovascular disease	A+T-	x	
5	E3/E4	No	Male	88	17	Alzheimer's disease possible	Int ADNC	A+T-	x	
6	E3/E4	No	Male	85	8	Mild cognitive impairment	Int ADNC	A+T-	x	x
7	E3/E4	Yes	Male	78	18	Frontotemporal dementia-NOS	High ADNC	A+T-	x	x
8	E3/E3	No	Male	85	16	Alzheimer's disease probable	High ADNC	A+T+	x	
9	E3/E3	Yes	Male	82	9	Alzheimer's disease possible	High ADNC	A+T+	x	x
10	E3/E4	No	Male	86	6.5	Alzheimer's disease probable	High ADNC	A+T+	x	
11	E3/E4	Yes	Male	60	12	Primary progressive aphasia, Logopenic variant	High ADNC	A+T+	x	x
12	E3/E4	No	Male	71	4.5	Alzheimer's disease probable	High ADNC	A+T+	x	
13	E3/E4	No	Male	54	18	Alzheimer's disease probable	High ADNC	A+T+	x	
14	E4/E4	No	Male	74	18	Alzheimer's disease probable	High ADNC	A+T+	x	
15	E4/E4	Yes	Male	61	11	Alzheimer's disease probable	High ADNC	A+T+	x	x
	E3/E3	Yes	Female	89+	7	Alzheimer's disease probable	High ADNC			x
	E3/E3	Yes	Female	87	4	Amyotrophic lateral sclerosis	High ADNC, Amyotrophic lateral sclerosis			x
	E3/E3	Yes	Male	64	16.5	Alzheimer's disease probable	High ADNC			x
	E3/E3	Yes	Female	66	6	Schizophrenia	Low ADNC			x
	E3/E3	Yes	Female	76	23	Alzheimer's disease probable	High ADNC			x
	E3/E3	Yes	Female	68	9	Alzheimer's disease probable	High ADNC			x
	E3/E3	Yes	Female	83	20	Dementia with Lewy bodies	High ADNC, Cerebrovascular disease			x
	E3/E4	Yes	Female	80	7	Alzheimer's disease probable	High ADNC			x
	E3/E4	Yes	Male	89+	8	Alzheimer's disease probable, Schizophrenia	Low ADNC			x
	E3/E4	Yes	Male	77	9	Alzheimer's disease probable	High ADNC			x
	E3/E4	Yes	Male	71	20	Alzheimer's disease probable	High ADNC, Amygdala-predominant Lewy body disease			x
	E3/E3	No	Female	76	10	Alzheimer's disease probable	High ADNC			x
	E3/E3	No	Female	83	10	Alzheimer's disease probable	High ADNC			x
	E3/E3	No	Female	87	16	Alzheimer's disease probable	High ADNC			x
	E3/E3	No	Male	62	12	Frontotemporal degeneration, behavioral variant	High ADNC			x
	E3/E3	No	Male	86	17	Alzheimer's disease probable	High ADNC			x
	E3/E3	No	Male	82	39	Dementia-NOS	High ADNC			x
	E3/E3	No	Male	64	4	Dementia with Lewy bodies	High ADNC			x
	E3/E3	No	Male	64	22	Alzheimer's disease probable	High ADNC			x
	E3/E3	No	Male	60	3	Dementia with Lewy bodies	High ADNC, Amygdala-predominant Lewy body disease			x
	E3/E3	No	Female	68	22	Alzheimer's disease probable	High ADNC, Amygdala-predominant Lewy body disease			x
	E3/E4	No	Male	78	17	Alzheimer's disease probable	Int ADNC			x
	E3/E4	No	Male	77	4	Frontotemporal degeneration, behavioral variant	High ADNC			x
	E3/E4	No	Male	81	18	Alzheimer's disease probable	High ADNC			x
	E3/E4	No	Male	78	6.5	Alzheimer's disease probable	High ADNC, Cerebrovascular disease			x
	E3/E4	No	Male	82	3	Alzheimer's disease probable	High ADNC			x

	E3/E4	No	Male	89+	4	Alzheimer's disease probable	High ADNC			x
	E3/E4	No	Female	89+	14	Alzheimer's disease probable	High ADNC, Cerebrovascular disease			x
	E3/E4	No	Female	80	12	Alzheimer's disease probable	High ADNC			x
	E3/E4	No	Female	79	4	Alzheimer's disease probable	High ADNC, Amygdala-predominant Lewy body disease			x
	E3/E4	No	Male	78	3	Alzheimer's disease probable	High ADNC, Cerebrovascular disease			x
	E3/E4	No	Male	71	4	Alzheimer's disease probable	High ADNC			x
	E4/E4	No	Female	76	6.5	Alzheimer's disease probable	High ADNC			x
	E4/E4	No	Male	59		Alzheimer's disease probable	High ADNC			x
	E4/E4	No	Female	83	3.5	Vascular dementia	High ADNC			x
	E4/E4	No	Female	83	16	Alzheimer's disease probable	High ADNC			x
	E4/E4	No	Male	64	5	Alzheimer's disease probable	High ADNC			x
	E4/E4	No	Male	78	12	Frontotemporal degeneration-NOS	High ADNC			x
	E4/E4	No	Male	59	33	Alzheimer's disease probable	High ADNC			x
	E4/E4	No	Male	67	10	Corticobasal degeneration	High ADNC			x
	E4/E4	No	Male	64	18	Alzheimer's disease probable	High ADNC			x
	E4/E4	No	Male	77	17	Mild cognitive impairment	High ADNC			x
	E2/E4	No	Male	66	20	Neurologically normal	Primary age-related tauopathy			x
	E3/E3	No	Male	81	5	Schizophrenia, Dementia-NOS	Low ADNC			x
	E3/E3	No	Male	83	18	Schizophrenia	Primary age-related tauopathy			x
	E3/E3	No	Male	84	20	Schizophrenia	Low ADNC			x
	E3/E3	No	Male	76	4	Neurologically normal	Low ADNC			x
	E3/E4	No	Male	89	12	Cognitively impaired	Low ADNC			x

Table S2. Number of nuclei before and after quality control (QC) filtering

SAMPLE ID	1	2	3	4	5	6	7	8	9	10	11	12	13	14	15	SUM
BEFORE QC	11084	11849	6936	12026	8901	11396	9405	13717	6422	11674	5526	8425	12182	5658	11236	146437
AFTER QC	10894	11455	6893	10492	8550	10758	6390	13649	4849	11225	2838	7586	11193	3476	10991	131239

Table S3. Number of nuclei by cell types across samples

SAMPLE ID / CELL TYPE	1	2	3	4	5	6	7	8	9	10	11	12	13	14	15	SUM
ASTROCYTE	572	1062	1994	428	593	1027	1224	1505	1872	1426	584	855	823	527	1191	15683
ENDOTHELIAL	26	27	31	5	18	15	46	35	25	40	21	85	23	16	31	444
INHIBITORY NEURON	1202	918	1345	1370	1408	983	370	1058	1118	624	158	471	389	69	803	12286
EXCITATORY NEURON	2628	1796	4602	3570	4526	3570	1483	4812	2690	3239	844	1849	933	310	2324	39176
MICROGLIA	289	26	552	71	336	260	222	357	226	688	110	183	263	64	335	3982
OLIGODENDROCYTE	4931	5488	955	649	915	3938	2392	4584	3964	3622	1246	3338	1717	1296	5147	44182
OLI PROGENITOR	409	518	710	305	431	388	430	303	633	594	373	503	549	288	419	6853
NONE	837	1620	303	495	323	577	223	995	665	992	140	302	152	268	741	8633
SUM	10057	9835	10189	6398	8227	10181	6167	12654	10528	10233	3336	7284	4697	2570	10250	122606

Table S4. Number of nuclei in microglia subpopulations across samples

SUBPOPULATIONS \ SAMPLE ID	1	2	3	4	5	6	7	8	9	10	11	12	13	14	15	SUM
HOMEOSTATIC	173	2	3	0	1	10	0	28	0	6	0	0	3	0	3	229
MOTILE	6	7	0	2	3	47	143	111	119	320	35	94	14	45	201	1147
ARM	9	7	1	362	255	127	8	46	96	98	10	30	43	22	5	1119
DYSTROPHIC	0	0	10	112	17	11	20	5	29	52	4	5	0	13	0	278
OLI_MICROGLIA	81	9	15	8	10	44	24	82	12	121	11	35	134	8	96	690
AST_MICROGLIA	5	1	6	21	3	3	15	19	1	13	4	7	12	9	8	127
EX_MICROGLIA	15	0	36	47	47	18	12	66	6	78	0	12	20	13	22	392
SUM	289	26	71	552	336	260	222	357	263	688	64	183	226	110	335	3982

Table S5. Unadjusted p-values of the differences across microglia subtypes using multivariate data change point tests

**P values**	Motile	ARM	Dystrophic
Homeostatic	0.0010	0.0010	0.0090
Motile		0.0050	0.0010
ARM			0.0010

Table S6. Antibody list

Antibody Name	Clone	Concentration	Host	Source	Catalog #
Anti-ferritin light chain	Polyclonal	1:30K (IHC)	Rabbit	Abcam	ab69090
Iba1	Polyclonal	1:1000 (IHC)	Rabbit	Wako	019-19741
CD163	Monoclonal (10D6)	1:100 (IHC)	Mouse	Abcam	ab201461
NAB228	Monoclonal	1:40K (IHC)	Mouse	CNDR, Univ of PA	
PHF1	Monoclonal	1:2000 (IHC)	Mouse	Gift from Dr. Peter Davies	
PU.1	Monoclonal (7C6B05)	1:50 (IHC)	Mouse	Biolegend	658002
CX3CR1	Monoclonal (8E10.D9)	1:200 (IHC)	Mouse	BioLegend	824001
FGD4	Polyclonal	1:100 (IHC)	Rabbit	Novus	NBP1-85532

Data S1. The DE gene lists of ARM, motile and dystrophic vs homeostatic

Data S2. The DE gene lists of ARM, motile and dystrophic, and homeostatic (one versus rest)

Data S3. GO enrichment analysis in Reactome pathways between ARM and dystrophic vs homeostatic.

Data S4. GO enrichment analysis in Biological Processes ontology between ARM, motile, dystrophic and homeostatic (one versus rest)

Data S5. The DE gene lists of ARM, Motile, and Dystrophic microglia using MetaCell data and significant ANOVA genes used in CSNs
